# Soft Rehabilitation Actuator With Integrated Post-stroke Finger Spasticity Evaluation

**DOI:** 10.3389/fbioe.2020.00111

**Published:** 2020-02-28

**Authors:** Ho Lam Heung, Zhi Qiang Tang, Xiang Qian Shi, Kai Yu Tong, Zheng Li

**Affiliations:** ^1^Department of Biomedical Engineering, Faculty of Engineering, The Chinese University of Hong Kong, Shatin, Hong Kong; ^2^Department of Surgery, The Chinese University of Hong Kong, Shatin, Hong Kong

**Keywords:** stroke, finite element method, finger spasticity, soft-elastic composite actuator, elastomer 3D printing

## Abstract

Strokes cause severe impairment of hand function because of the spasticity in the affected upper extremities. Proper spasticity evaluation is critical to facilitate neural plasticity for rehabilitation after stroke. However, existing methods for measuring spasticity, e.g. Modified Ashworth Scale (MAS), highly depends on clinicians’ experiences, which are subjective and lacks quantitative details. Here, we introduce the first rehabilitation actuator that objectively reflects the condition of post-stroke finger spasticity. The actuator is 3D printed with soft materials. By considering the finger and the actuator together, the spasticity, i.e. stiffness, in finger is obtained from the pressure–angle relationship. The method is validated by simulations using finite element analysis (FEA) and experiments on mannequin fingers. Furthermore, it is examined on four stroke subjects and four healthy subjects. Results show the finger stiffness increases significantly from healthy subjects to stroke subjects, particularly those with high MAS score. For patients with the same MAS score, stiffness variation can be a few times. With this soft actuator, a hand rehabilitation robot that may tell the therapeutic progress during the rehabilitation training is readily available.

## Introduction

Stroke has been the leading cause of disability. Every 40 s there will be a new stroke case (Emelia et al., 2017). Finger flexor spasticity, a motor disorder which results from impaired reflex function and causes involuntary muscle contraction, is one of the common disabling symptoms after stroke (Dietz and Sinkjaer, 2007). The onset of spasticity can occur within the first few days or weeks to around 30% of patients (Mayer and Esquenazi, 2003). The hands of patients who are affected by significant levels of post-stroke flexor spasticity remain tightly clenched due to the increased muscle tone in finger flexors, which is also believed to be the underlying reason of the difficulty in extending the fingers for stroke patients (Marciniak, 2011). In other words, this spasticity increases stiffness of the finger joints and furthermore leads to a decrease of their range-of-motion (ROM), creating severe reduction to hand function (Sadarangani et al., 2017). As spasticity and motor recovery are both related to neural plasticity after stroke, to lead targeted rehabilitation interventions, it is suggested that clinicians should decide the best treatment option for each patient based on their spasticity condition (Hong et al., 2018; Jeanette et al., 2019). In current clinical practice, primary measures (Bohannon and Smith, 1987; Copley and Kuipers, 1999; Gregson et al., 1999; Mackey et al., 2004) such as Modified Ashworth Scale (MAS), Modified Tardieu Scale (MTS), Tone Assessment Scale, and King’s Hypertonicity Scale are widely used to grade the resistance on the joints during passive soft tissue stretching (Thibaut et al., 2013). Such clinical scores quickly generate insights for therapists about the change in passive stiffness opposing the rotation of the examined joints. Nevertheless, result subjectivity and rater reliability have been continuously questioned by researchers since the measurement is completely dependent on clinicians’ experience (Damiano et al., 2002; Fleuren et al., 2010). Consistency of the results cannot be ensured among different assessors even if adequate training is provided (Pandyan et al., 1999). Therefore, there is a need of a procedure to objectively quantify finger joint stiffness for assessing hand function after stroke.

Various simple mechatronic devices are designed for standalone finger joint stiffness measurement after stroke (Milner and Franklin, 1998; Kamper and Rymer, 2000; Angelo et al., 2006; Yap et al., 2017). During the measurement, the forearm of patients is vertically clamped to a table for coupling the rotation of metacarpophalangeal (MCP) joints of all digits to a servomotor. To compute the joint stiffness, the angle and torque of MCP joints are measured by a torque transducer during rotation. However, since the stiffness of MCP joint cannot be individually assessed across each digit, it remains difficult to systematically evaluate the impairment of hand function based on the condition of each finger. Furthermore, due to the bulky size of all components (mounting platform of forearm, servomotor, and torque transducer), the devices are hard to be used to further examine the stiffness of other joints, e.g. proximal interphalangeal (PIP) joints that the stiffness is comparable to MCP joints after contracture (Prosser, 1996; Tang et al., 2019), in each finger. Additional robotic systems are required to independently examine the joint contracture for each digit in order to standardize the quantification of the hand function impairment level for better interventions guidance.

Recently, robotic hands have been proposed as wearable devices that facilitate movement of each individual finger in both flexion and extension direction. Susanto et al. have introduced the hand exoskeleton robot for active individual finger control via joint moment sensing (Tong et al., 2010; Evan et al., 2015). Therapeutic effect of the device has been demonstrated in the post-stroke rehabilitation training. To reflect the improvement of hand function, angle and torque of MCP and PIP joints are measured across each individual finger for examining the finger individuation (Wolbrecht et al., 2018), which is an important and comprehensive target for rehabilitative hand training. One major limitation of these robotic hands is shown in the bulkiness of mechanical linkages that translate finger movement to linear actuators, making them unsuitable for patients wearing them to perform activities of daily living (ADL). To address this problem in the rigid mechanical robotic systems, soft robots have been developed (Laschi and Cianchetti, 2014). Yap et al. (2017) and Cappello et al. (2018) have developed the robotic hands by the utilization of soft bending actuators to their bi-directional robotic gloves (Hong et al., 2017), which further reduced their weight and facilitated the performance of ADL, e.g. grasp of a bottle, using the soft robotic gloves. Nevertheless, since the evaluation of finger joint stiffness is not integrated with these actuators, the continuous stiffness measurement to indicate the performance of hand function using existing soft robotic hands is still lacking, and only pre-determined training exercises can be offered to patients regardless of their finger spasticity conditions. Since the level of finger spasticity varies during the ADL and therapeutic training, it is important to know the finger joint stiffness in real-time (Thibaut et al., 2013). By integrating the function of individual joint stiffness sensing to rehabilitation training, the robotic system could therefore offer optimum training exercises and assistance with ADL to stroke patients.

**FIGURE 1:**
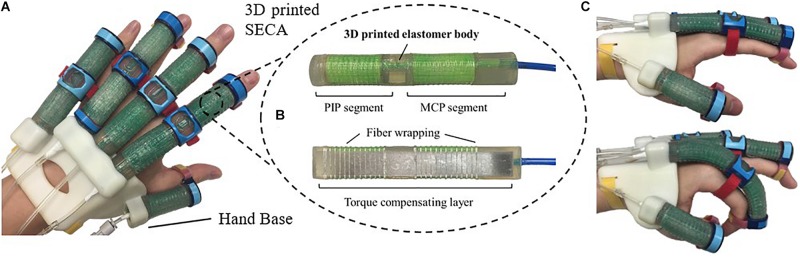
**(A)** Prototype of the 3D printed soft robotic hand for stroke rehabilitation and assistance of ADL. **(B)** Prototype of the 3D printed SECA. **(C)** Flexion and extension of the index finger (MCP and PIP joints) at 120 kPa on pneumatic actuation.

In this study, we propose the 3D printed soft-elastic composite actuator (SECA) that adopted to our soft robotic hand (Figure 1A) for facilitating both flexion and extension of spastic fingers during the performance of ADL and rehabilitation training (Heung et al., 2019b). SECA modeling is presented for the relationship between the input pressures and the bending angles according to the energy distribution inside the SECA and the spastic finger joints. Stiffness equations of spastic finger joints are derived from the pressure–angle relationship of SECA when worn on spastic fingers. In the experimental results, the bending angles of SECA with free bending and when placed on model compromised fingers are given, and the stiffness of the model fingers is further examined by the derived stiffness equations upon the actuation of 3D printed SECA, as well as finite element method (FEM) and analytical results for validation. Last, eight subjects (four in stroke and four in healthy condition) are recruited for the preliminary evaluation with the 3D printed SECA installed on index fingers, and the results of measured MCP and PIP joint stiffness are compared with the scores of MAS for reflecting the clinical potential of our method.

## Significance

Stroke causes severe impairment of hand function. Spasticity and motor recovery are both related to neural plasticity after stroke. For rehabilitation planning, the level of finger spasticity that varies over different hand function tasks must be accurately evaluated. Unfortunately, existing spasticity indicators lack reliability, objectivity, consistency, and quantitative stiffness details. Moreover, there exists no hand rehabilitation devices that help objectively evaluate finger spasticity. Here, we introduce the first soft actuator for hand rehabilitation that quantifies spasticity of impaired fingers, i.e. stiffness, in real time. The method is validated with phantom fingers and eight subjects. With the stiffness information, optimal training tasks may be offered by our soft robotic system according to the varying spasticity conditions in fingers. Training outcome may also be improved by indicating the timely therapeutic progress, i.e. the decrease of joint stiffness, to patients for motivating them to achieve better hand function improvement.

## 3D Printed Soft Actuator

The 3D printed SECA (Figure 1B) presented in this work is composed of an elastomeric actuator body, fiber wrapping, and torque compensating layer, similar to our previous design by silicone molding (Heung et al., 2019b). The difference between SECA and traditional fiber-reinforced bending actuator (FRA) is that the SECA has a composite design with a torque compensating layer that can facilitate both flexion and extension on the same unit. Flexion and extension by the SECA are controlled under pressurization and depressurization, respectively. Meanwhile, FRA allows flexion under pressurization with a bottom strain limiting layer. Extension of FRA under depressurization can only be passively driven by the limited elasticity of elastomeric actuator body.

Metacarpophalangeal and PIP segments on the SECA correspond to actuation of the MCP and PIP joints of the human finger. When pressurized, the fiber wrapping around the actuator surface suppresses radial expansion, and the torque compensating layer further eliminates any axial elongation at the bottom, leaving only the upper section of the actuator to be elongated for bending the fingers. When depressurized, the torque compensating layer provides an assistive bending moment for the extension of fingers to the initial position (Figure 1C). The SECA weighs 21 g, which reduced the overall moment of inertia upon wearing and facilitated a more natural movement to fingers. Distal interphalangeal (DIP) joint is not addressed at all due to its small contribution to daily activities (around 15% of a functional grip) (Leibovic and Bowers, 1994).

In this study, we introduce the use of the latest industrial silicone 3D printer (ACEO Imagine, Burghausen, Germany) developed by ACEO^®^ – WACKER Chemie AG, to directly 3D print the SECA. In the design of our 3D printed SECA, we select ACEO Silicone GP Shore A 30, an available 3D printed silicone rubber offered by ACEO^®^ – WACKER Chemie AG, as the printing material for the elastomeric actuator body. The 3D printed silicone rubber offers sufficient elongation (450%) and tensile strength (6 MPa) to support large deformation and withstand high input pressure without creating any rupture, while the hardness (Shore A 30) is comparable to the silicone rubber of Dragon Skin Series (2019). Furthermore, a thin rigid elastic plate (A2 stainless steel plate) is used for the torque compensating layer, and directly reinforced to the bottom of SECA by the fiber wrapping.

## Modeling of the SECA

### Free Space Bending

In a bending state, assume that the energy loss to the surroundings is negligible, work done by the input air pressure in the chambers is equal to the bending strain energy stored in the elastomer body and torque compensating layer (Wang et al., 2018). Therefore, the bending angle to the input air pressure can be found by the conservation of energy (Supplementary Material).

(1) $$W_{P}=\left(W_{A}+W_{L}\right)$$

in which *W*_*P*_, *W*_*A*_, and *W*_*L*_ denote the work done by input air pressure and the bending strain energy stored in the 3D printed actuator body and the torque compensating layer, respectively.

**FIGURE 2:**
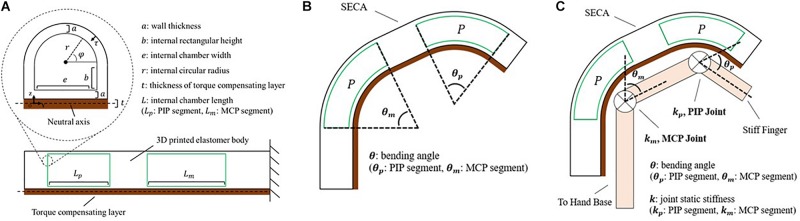
Modeling of the soft-elastic composite actuator. **(A)** Closeup view: cross-sectional area of the distal tip of SECA showing the defined variables. Lower center: section view from lateral direction displaying the chamber length of SECA. **(B)** Illustration of the 3D printed SECA bending in free space. **(C)** Bending of the 3D printed SECA considering stiff finger joints.

Because the SECA bends due to the work done created by the internal pressure acting on the chamber, we equate this work done (Supplementary Material).

(2) $$W_{P}=P\,\Delta \,V$$

where *P* is the input pressure, θ is the bending angle, Δ*V* is the increase of volume (Figure 2A) calculated by:

(3) $$\Delta V=(\int_{0}^{r}2\sqrt{r^{2}-z^{2}}\left(\frac{t}{2}+a+b+z\right)\text{d}z+\int_{0}^{b}e\left(\frac{t}{2}+a+z\right)\text{d}z)\theta$$

with the bending strain energy stored inside the 3D printed actuator body (Supplementary Material).

(4) $$W_{A}\approx \int_{0}^{a}\int_{0}^{\frac{\pi }{2}}2\,w_{m}\,L\,\left(a+b+\left(r+\tau \right)\,\sin\phi \right)\,\text{d}\phi \,\text{d}\tau$$

where *L* is the actuator length, dτ is the differential wall thickness element, dφ is the circumferential angle element (Figure 2A), *w*_*m*_ is the strain energy density function of silicone rubber described by an Ogden 2-Parameter model (Ogden, 1984) as:

(5) $$w_{m}=\sum_{n=1}^{2}\frac{\mu_{n}}{\alpha_{n}}\,\left(\lambda^{\alpha_{n}}+\lambda^{-\alpha_{n}}-2\right)$$

where λ is the principle stretch, material coefficient α_1_ and α_2_ are the strain hardening exponents, μ is the small strain shear modulus, μ_1_ and μ_2_ are defined by:

(6) $$2\,\mu =\mu_{1}\,\alpha_{1}+\mu_{2}\,\alpha_{2}$$

and inside the torque compensating layer (Supplementary Material).

(7) $$W_{L}=\frac{E\,I\,\theta^{2}}{2\,L}$$

where*E* is the Young’s modulus, *I* is the second moment of area, and *EI* is the flexural rigidity of torque compensating layer (Turcotte and Schubert, 2002). From the equation, there will be no significant difference between the torque compensating layer and the strain limiting layer from traditional fiber-reinforced actuator (FRA) if the flexural rigidity is too small (Heung et al., 2019b).

Solving *W*_*P*_ = *W*_*A*_ + *W*_*L*_ yields an expression between the bending angle and input pressure as (Figure 2B and Supplementary Material).

(8) $$P=\frac{2\,L\,\int_{0}^{a}\int_{0}^{\frac{\pi }{2}}2\,w_{m}\,L\,\left(a+b+\left(r+\tau \right)\,\sin\phi \right)\,\text{d}\phi \,\text{d}\tau +E\,I\,\theta^{2}}{2\,L\,\Delta \,V}$$

### Constrained Bending on Model Fingers

To estimate the finger joint stiffness using the 3D printed SECA, work done of finger joints at different angular position is considered within the energy system, which becomes (Supplementary Material).

(9) $$W_{P}=\left\{ \begin{array}{c} W_{A}+W_{L}-W_{\mathrm{Joint}},\theta \in \left[0,\theta_{0}\right)\\ W_{A}+W_{L},\theta =\theta_{0}\\ W_{A}+W_{L}+W_{\mathrm{Joi}\,n\,t},\theta \in \left(\theta_{0},90^{\circ }\right] \end{array}\right.$$

where θ_0_ is the resting angle of the joint and always less than 90°, *W*_*Joint*_ is the elastic potential energy (work done) of the finger joints. Excluding the dynamics associated with the fingers, energy stored in the joints is given by:

(10) $$W_{\text{Joint}}=\frac{1}{2}\,k\,{\left(\theta -\theta_{0}\right)}^{2}$$

where *k* is the joint stiffness (Figure 2C; Woods and Lawrence, 1997). To treat the finger joint angle to be the same as the bending angle of 3D printed SECA, the pressure–angle relationship of 3D printed SECA on the fingers is presented by Supplementary Material.

(11) $$P=\left\{ \begin{array}{c} \frac{2\,\left(W_{A}+W_{L}\right)-k\,{\left(\theta -\theta_{0}\right)}^{2}}{2\,\Delta \,V},\theta \in \left[0,\theta_{0}\right)\\ \frac{W_{A}+W_{L}}{\Delta \,V},\theta =\theta_{0}\\ \frac{2\,\left(W_{A}+W_{L}\right)+k\,{\left(\theta -\theta_{0}\right)}^{2}}{2\,\Delta \,V},\theta \in \left(\theta_{0},90^{\circ }\right] \end{array}\right.$$

### Joint Stiffness Estimation

When there is no exerted voluntary movement, our fingers tend to curl inward and remain in a flexed position (θ_0_) due to the muscle tone naturally presented in finger flexors (e.g., flexor digitorum profundus) being larger than that of finger extensors (e.g., extensor digitorum) (Wehbe and Hunter, 1985; Loh et al., 2018). Passive extension of the fingers (i.e. θ ∈ [0,θ_0_]) from flexed position would stretch the flexor muscles, creating resistance at the joints to oppose the movement. For stroke patients, strong resistance would be induced against extension due to the excess tone in finger flexors, called hypertonia (Fève et al., 1998). In this section, we quantify the resistance due to hypertonia in terms of the joint stiffness using the analytical model of SECA.

Since the major design consideration is for stroke rehabilitation, only the joint stiffness upon extending the fingers is of our interest. Further flexion of the fingers after resting angle (i.e. θ∈[θ0, 90]∘)would not be considered into stiffness estimation. Previously, it has been proved that the stiffness of finger joints can be treated as constant values when they are hold in static position (Kamper and Rymer, 2000; Kamper et al., 2003; Brokaw et al., 2011; Claudia et al., 2018). Hence, to begin with, rearranging the analytical model in the region of θ∈[0,∘θ0), the joint stiffness equation is:

(12) $$k=\frac{2\,L\,\left(\int_{0}^{a}\int_{0}^{\frac{\pi }{2}}2\,w_{m}\,L\,\left(a+b+\left(r+\tau \right)\,\sin\text{φ}\right)\,\text{d}\text{φ}\,\text{d}\tau -P\,\Delta \,V\right)+E\,I\,\theta^{2}}{L\,{\left(\theta -\theta_{0}\right)}^{2}}$$

Near the singularity (i.e. only small difference between the bending angle of SECA θ and the resting angle of finger joint θ_0_), accuracy of the results would be significantly affected. Therefore, it is crucial to define the possible ranges of MCP and PIP joint angles and input pressures for the joint stiffness equation (Eqs 12) as:

(13) $$\theta_{m}\in \left[0,\gamma \,\theta_{0\,\_\,m}\right]\,\mathrm{and}\,\theta_{p}\in \left[0,\gamma \,\theta_{0\,\_\,p}\right],0<\gamma <1$$

(14) $$P=\text{cutoff}\mathrm{when}\left(\theta_{m}>\gamma \theta_{0\,\_\,m}\mathrm{or}\theta_{p}>\gamma \theta_{0\,\_\,p}\right)$$

where γ is an empirical coefficient chosen to be 0.7 to avoid reaching singularity. In the bending state of the 3D printed SECA, cutoff pressure is defined as soon as the measured MCP or PIP joint angle exceeds its upper limit (γθ_0_*m*_ or γθ_0_*p*_), and therefore the SECA is not further actuated and influenced by the singularity in the model.

## Free Space Bending Angles Measurement

**FIGURE 3:**
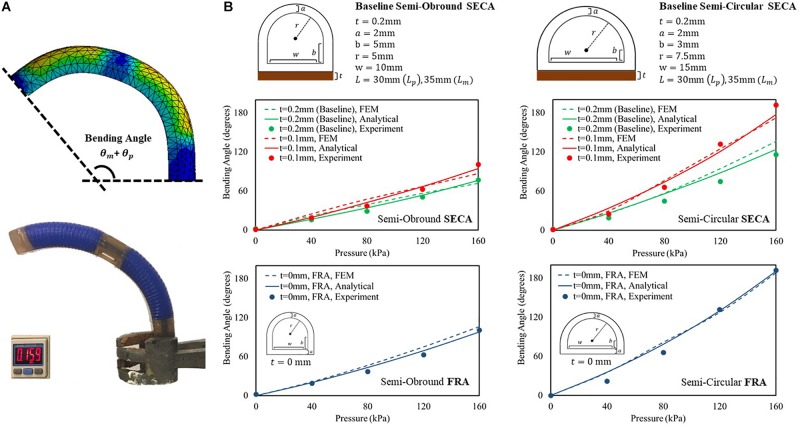
**(A)** FEM-simulated and experimental bending behavior of 3D printed baseline semi-circular SECA at 160 kPa of input pressure. **(B)** Free space bending pressure–angle relationship of semi-obround and semi-circular SECAs with baseline geometrical parameters and varying thickness of torque compensating layers [0.1 mm, 0 mm (strain limiting layer for FRA)].

Prior to the experiments of 3D printed SECA on spastic fingers, we examine the accuracy of described energy distribution in the actuator by comparing the bending angles in free space. The analytical models in free space bending are then well-validated experimentally and by FEM (Figure 3A and Supplementary Video S1). Two baseline sets of geometrical parameters (Figure 3B) are chosen for the 3D printed semi-obround and semi-circular SECAs (Supplementary Figure S4). Additionally, two variations of the thickness of torque compensating layers are also applied (0.1 mm, 0 mm that represents the strain limiting layer for FRAs) in the analytical model (Panagiotis et al., 2015; Fionnuala et al., 2017). For the FE model, Ogden 2-Parameter model with coefficient μ_1_ = 0.027106 MPa, α_1_ = 4.2304, μ_2_ = 9.2012 MPa, α_2_ = 0.041832 is determined by the uniaxial tensile test of the 3D printed silicone samples (Supplementary Figure S1). Gravity effect is minimized by the torque compensating layer, and therefore the bending angles will not be significantly affected. Maximum input pressure supplied to the SECA is limited to 160 kPa.

To consider the sum of PIP and MCP segment angles (θ_*p*_ + θ_*m*_) as the bending angle of the 3D printed SECA, we can conclude the correct description of energy distribution inside the SECA based on the observed matching between model, FEM, and experiment results (Figure 3B and Supplementary Material). However, it is worth to note that if the thickness of torque compensating layer decreases to 0.1 mm or smaller, the flexural rigidity of the layer will be too small that no significant difference can be indicated between SECA and traditional FRA, e.g. at 160 kPa, the same experimental bending angle of 101° from the baseline semi-obround SECA and FRA, and 192° from the baseline semi-circular SECA and FRA. Similar trends have also been confirmed by the analytical modeling and FEM simulation of 3D printed SECA (Supplementary Material). In such case, the SECA would become difficult to flex and extend the spastic fingers during rehabilitation. Therefore, the layer thickness is a crucial factor in the functional SECA design (layer thickness at least 0.2 mm or larger) at the beginning before considering further application of joint stiffness estimation.

## Constrained Bending Angles Measurement With Stiffness Estimation

**FIGURE 4:**
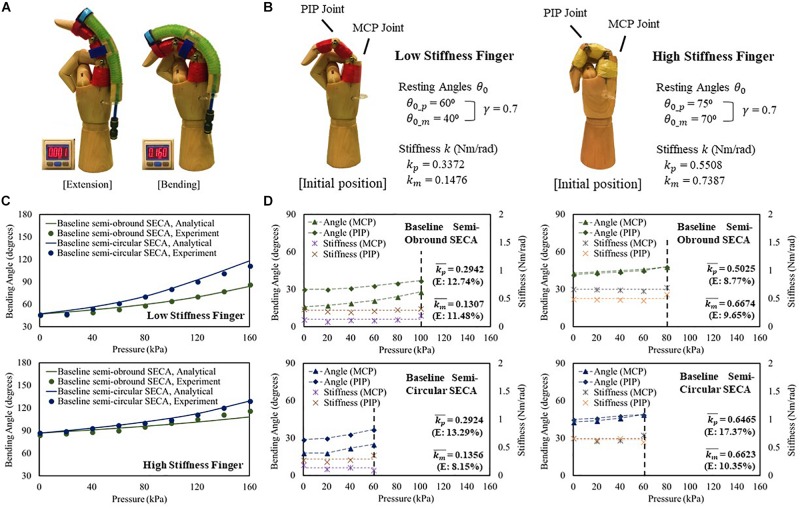
**(A)** Constrained flexion and extension of low stiffness finger with 3D printed baseline semi-circular SECA. **(B)** Characteristic of the model low and high stiffness fingers. **(C)** Constrained bending pressure–angle relationship of baseline semi-obround and semi-circular SECAs on high and low stiffness fingers. **(D)** Estimated stiffness of MCP and PIP joints and the corresponding angles measured on high and low stiffness fingers with baseline semi-obround and semi-circular SECAs.

To study the SECA on fingers (Figure 4A and Supplementary Video S1), two mannequin hands in which the index fingers are installed with high stiffness (*k*_*p*_ = 0.5508 Nm/rad, *k*_*m*_ = 0.7387 Nm/rad) and low stiffness (*k*_*p*_ = 0.3372 Nm/rad, *k*_*m*_ = 0.1476 Nm/rad) torsion springs at the PIP and MCP joint positions are designed to model the impaired fingers (Figure 4B). Baseline semi-obround and semi-circular SECAs are tested in this section. A guideline is provided for proper installation of the SECA to the finger to ensure consistency with each use (Supplementary Material).

For both low and high stiffness fingers, the model results agree with the experiment results (Figure 4C). A maximum difference of 8.67° of bending angle is observed on high stiffness finger with baseline semi-obround SECA at 160 kPa. Although satisfactory results are demonstrated, it is often that in real situations, finger joint stiffness is an unknown variable, e.g. the flexed fingers in stroke patients. Here, we can predict the stiffness based on the individual measured MCP and PIP joint angles at different input pressures (Figure 4D). As mentioned, γ = 0.7 is chosen for the upper limits of measured MCP and PIP joint angles. The end of the actuation of 3D printed SECAs is indicated when the angle is out of range. Eventually, the MCP and PIP joint stiffness is taken by the average of stiffness values from different input pressures (Figure 4D and Supplementary Material). Compared with the design specification of the model fingers (Figure 4B), a mean error of 0.027 Nm/rad is observed considering all eight sets of estimated stiffness values, while a maximum difference between the original and estimated result is seen on the PIP joint of high stiffness finger with baseline semi-circular SECA (error of 0.0957 Nm/rad).

## Preliminary Evaluation of Post-Stroke Finger Joint Stiffness on Stroke Patients

**FIGURE 5:**
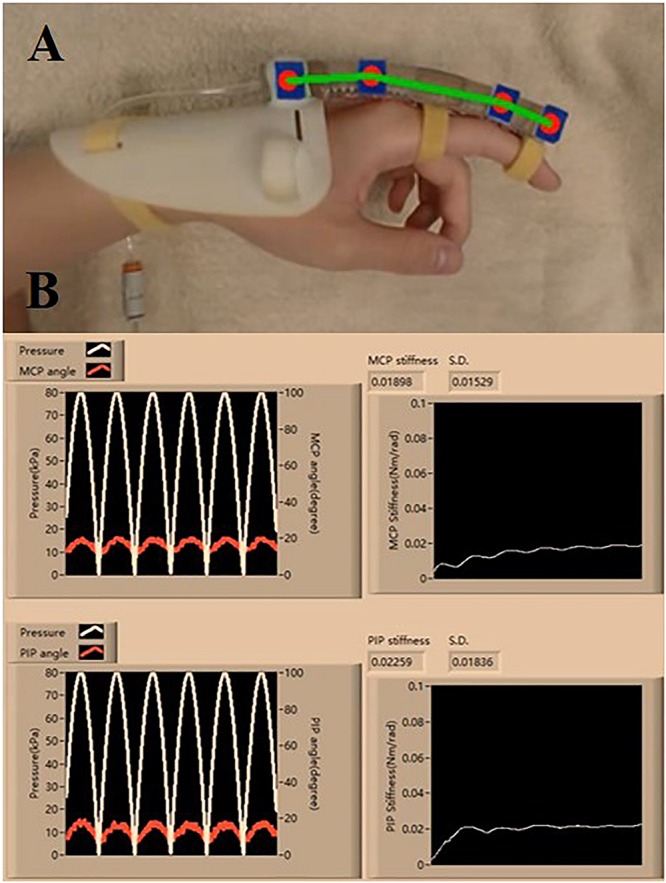
**(A)** Proper wearing of the SECA. **(B)** Estimation process of the MCP and PIP joint stiffness, and the actuated pressure and angle readings (six actuation cycles are set. Stiffness values are constantly calculated at different pressure points, and the final value is taken from the average of all the collected samples of stiffness).

The clinical evaluation is registered to the Joint Chinese University of Hong Kong-New Territories East Cluster (CUHK-NTEC) Clinical Research Ethics Committee (Ref. ID: NCT03286309). Baseline semi-circular SECA is selected due to the larger ROM generated on the impaired fingers during the measurement of MCP and PIP joint stiffness (Figure 4C and Supplementary Material). In hand rehabilitation after stroke, better functional recovery of spastic fingers can be facilitated with the rehabilitation devices that can generate sufficient ROM to assist finger flexion and extension (Panagiotis et al., 2013; Evan et al., 2015). The SECA is proximally attached to a hand base and secured to the index finger with Velcro straps (Figure 5A; Heung et al., 2019a). Actuation pressure and bending angles are recorded for calculating the joint stiffness (Figure 5B).

Four stroke subjects who have demonstrated weak hand strength with different levels of finger flexor spasticity are recruited (Supplementary Table VII). Four healthy subjects also participated in the study to serve as the control. All subjects have given their informed consents. For consistency, the whole upper limb remains in a neutral position during the measurement (Supplementary Material; Centers of Disease Control and Prevention, 2019). To ensure the accuracy of the estimated joint stiffness, the subjects must stay relaxed to not influence the bending performance of the 3D printed SECA.

A brief physical examination is performed on the subjects (Table 1). The degree of spasticity of the fingers is assessed by a trained clinical assessor who is unaware of the experimental condition. For healthy subjects, it is suggested that their condition can be classified as “no increase in flexor muscle tone” (MAS score = 0) (Kamper and Rymer, 2000).

**TABLE 1:** Clinical tone and estimated MCP and PIP joint stiffness.

Subject	Hemiplegic side	MAS score^1^ (finger)	PIP resting angle θ_0____*_*p*_* (°)	MCP resting angle θ_0____*_*m*_* (°)	PIP stiffness *k*_*p*_ (nm/rad)	MCP stiffness *k*_*m*_ (nm/rad)
S1	Left	1+	72	54	0.09240 (±0.017090)	0.08902 (±0.005416)
S2	Left	1+	48	35	0.08925 (±0.018371)	0.09995 (±0.011663)
S3	Left	3	90	61	0.75325 (±0.061346)	0.63123 (±0.070554)
S4	Left	1+	67	37	0.03673 (±0.004110)	0.14948 (±0.049062)
H1^2^	(Right)	0	44	46	0.00934 (±0.003048)	0.01728 (±0.012844)
H2^2^	(Left)	0	49	40	0.01086 (±0.005967)	0.03106 (±0.016772)
H3^2^	(Right)	0	39	58	0.01530 (±0.005091)	0.01000 (±0.006121)
H4^2^	(Left)	0	36	45	0.02583 (±0.022760)	0.01670 (±0.006590)

*^1^Ranging from 0 to 4 [0 = no increase in muscle tone, 1 = slight increase in muscle tone at the end of the ROM, 1+ = slight increase in muscle tone throughout less than half of the ROM, 2 = more marked increase in muscle tone throughout most of the ROM, 3 = considerable increase in muscle tone throughout most of the ROM, 4 = complete rigid of the affected joint(s)] (Ansari et al., 2008). Higher MAS score indicates severe spasticity (Kamper et al., 2006). Flexor tone is assessed for MAS. ^2^Control subjects. index finger on the dominant hand is chosen for the assessment.*

Quantification of MCP and PIP joint stiffness is conducted (Table 1 and Supplementary Videos S2, S3). γ = 0.7 is used for the experiments. For stroke patients, the joint stiffness values disclose a similar tendency with the spasticity levels measured by MAS. The results of subject S3 are clearly higher than that of the other subjects, reflecting more severe finger spasticity suffered by subject S3. Furthermore, when comparing the values with that of normal subjects, larger joint stiffness is observed on stroke subjects due to post-stroke hypertonia in finger flexors. The results are found to be near the stiffness ranges presented by existing studies of MCP and PIP joint stiffness (Kamper and Rymer, 2000; Dionysian et al., 2005) (Stroke: around 0.55 Nm/rad with MAS = 3, Healthy: around 0.03 Nm/rad), which would be indicative of the effects of any hand rehabilitation training, e.g. improved flexor spasticity to be reflected in the decrease of joint stiffness.

## Conclusion

In this article, we have demonstrated the methodology of applying the 3D printed SECA to finger stiffness evaluation using the analytical models. The accuracy of the models is validated both in free space and on model fingers. Preliminary results showing the joint stiffness of stroke and healthy subjects are obtained using the models, which are supportive to existing clinical measures. In the future, our proposed method can be generalized to a rehabilitation robotic hand that reflects the stiffness of each finger in real time. With the stiffness information, optimal training tasks may be planned for each stroke individual depending on the current finger spasticity condition. Therapeutic progress may also be indicated in detail to motivate patients for achieving better improvement during rehabilitation training.

## Data Availability Statement

All datasets generated for this study are included in the article/Supplementary Material.

## Ethics Statement

The studies involving human participants were reviewed and approved by the Joint Chinese University of Hong Kong-New Territories East Cluster (CUHK-NTEC) Clinical Research Ethics Committee (Ref. ID: NCT03286309). The patients/participants provided their written informed consent to participate in this study.

## Author Contributions

HH is the first author who developed the mathematical theories, designed the experimental devices and experimental protocols, implemented the data collection software, performed the experiments, analyzed the experimental results, and participated in the manuscript preparation. ZT and XS performed the experiments, contributed to the discussions and analysis, and participated in the manuscript revisions. KT and ZL are the corresponding authors who supervised the project, contributed to the discussions and analysis, and participated in the manuscript revisions. All authors read and approved the submitted version of the manuscript.

## Conflict of Interest

The authors declare that the research was conducted in the absence of any commercial or financial relationships that could be construed as a potential conflict of interest.
