# Supplementary material for: Soft Rehabilitation Actuator With Integrated Post-stroke Finger Spasticity Evaluation
Source: Front Bioeng Biotechnol. 2020 Feb 28;8:111. doi: 10.3389/fbioe.2020.00111 (PMC7059754; doi:10.3389/fbioe.2020.00111)
Supplement: Supplementary file 4 [file Presentation_1.PDF]

Supplementary Information for

Soft Rehabilitation Actuator with Integrated Post-Stroke Finger Spasticity Evaluation

Kelvin H.L. Heung [1], Zhiqiang Tang [1], Xiangqian Shi [1], Raymond K.Y. Tong [1] and Zheng Li [2]

[1] Department of Biomedical Engineering, the Chinese University of Hong Kong, Hong Kong

[2] Department of Surgery, the Chinese University of Hong Kong, Hong Kong

Address correspondence to:
Zheng Li
Chow Yuk Ho Technology Centre for Innovative Medicine
Department of Surgery
The Chinese University of Hong Kong
Shatin
Hong Kong
E-mail: lizheng@cuhk.edu.hk

Raymond K.Y. Tong
Department of Biomedical Engineering
The Chinese University of Hong Kong
Shatin
Hong Kong
E-mail: kytong@cuhk.edu.hk

Content

S1 Modeling of the Soft-Elastic Composite Actuator

S1.1 Finite Element Simulation

S1.2 Analytical modeling

S1.2.1 Free space bending

S1.2.2 Constrained Bending on Model Fingers

S1.2.3 Joint Stiffness Estimation

S2 Lab Experimental Validation

S2.1 Experimental Setup

S2.2 Free Space Bending Measurement

S2.3 Constrained Bending Measurement on Model Fingers

S2.3.1 Actuator Installation

S2.3.2 Bending Angles on model fingers

S2.3.3 Stiffness Estimation of the model fingers

S3 Preliminary Human Subject Evaluation

S3.1 Experimental Configuration

S3.2 Subjects' demographic information

S3.3 Results of the MCP and PIP joint stiffness

S1 Modeling of the Soft-Elastic Composite Actuator

In this section, we will explain the process of establishing the finite element simulation and analytical model to present the actuation of Soft-Elastic Composite Actuator.

S1.1 Finite Element Simulation

Uniaxial tensile test is conducted to the 3D printed silicone rubber (ACEO Silicone GP Shore A 30) with the manufacturer ACEO® - WACKER Chemie AG using the Zwick Universal Tester ProLine Z050TN (Zwick Roell Group, Ulm, Germany) based on ISO 37 standard. Three test specimens are 3D printed according to ISO 37 Type 2 protocol and stretched at a rate of 60 mm/min. Figure S1 shows the averaged stress-strain relationship of the 3D printed silicone rubber. The measured stress-strain data is then fitted into the Ogden 2-Parameter model on Hyperfit [1] with material constants of μ_1 = 0.027106 MPa, α_1 = 4.2304, α_2 = 9.2012 MPa, μ_2 = 0.041832.

3D FEM model is established on ANSYS Workbench for the Soft-Elastic Composite Actuator. A Static Structural analysis is performed to analysis the actuator bending angle on different input pressures. Settings of the model was already reported in our previous work [2]. Only simplification

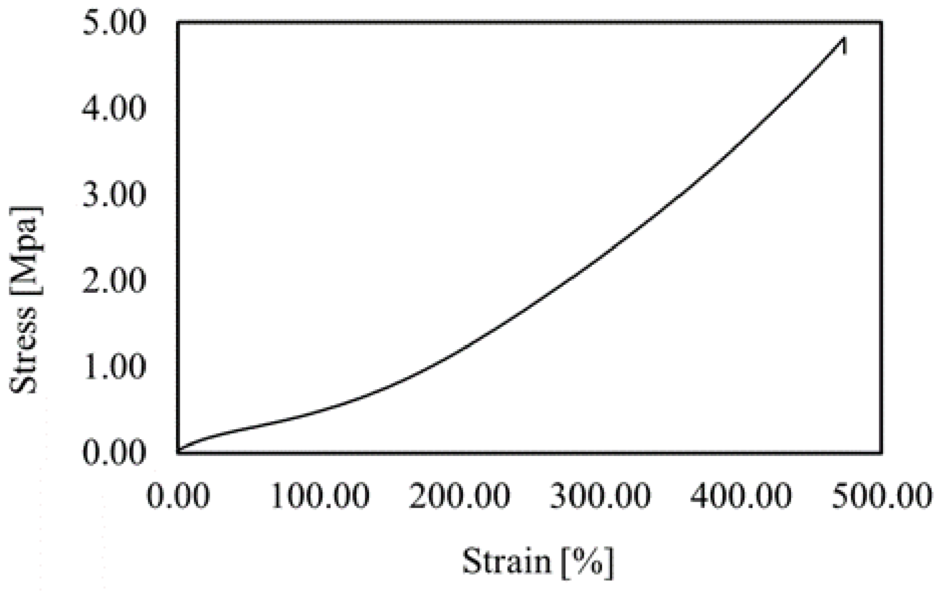

Fig. S1. Averaged stress-strain curve of the 3D printed silicone rubber (ACEO Silicone GP Shore A 30)

is kept neglecting the pressure inlets by directly applying pressure to the internal chamber walls. To create quality mesh for accurate results, 3D 10-Node tetrahedral structural solid elements (ANSYS element type SOLID187) and 3D 20-Node structural solid elements (ANSYS element type SOLID186) are used for the 3D printed actuator body and torque compensating layer respectively. For the fiber wrapping, 3D 2-Node beam elements (ANSYS element type BEAM188) are adopted to accommodate with the diameter of fiber.

S1.2 Analytical modeling

Assume that there is no energy loss to the surroundings, the quasi-static relationship between the input pressure and bending angle of the 3D printed Soft-Elastic Composite Actuator can be derived based on the law of conservation of energy, including the work done by input air pressure W_P, bending strain energy stored in the 3D printed actuator body W_A, bending strain energy stored in the torque compensating layer W_L, and work done of the finger joint W_{Joint}. In the following, we will describe the energy components individually for the construction of free space bending model and constrained bending model on finger joints. Then, to rearrange the models, we will obtain the analytical expressions that are applicable for finger joint stiffness estimation according to the input pressure and measured bending angle of Soft-Elastic Composite Actuator.

S1.2.1 Free space bending

The response of bending angle to the input air pressure can be found by the conservation of energy in the 3D printed Soft-Elastic Composite Actuator, which is represented by

$$W_P = (W_A + W_L) \tag{1}$$

Work done by input air pressure W_P. On pressurization, bending motion occurs on the MCP and PIP segment of Soft-Elastic Composite Actuator, and the volume of internal air chambers will be increased accordingly. Assume there is no cross-sectional deformation of the air chamber [3], the work done by the input pressure to the increased chamber volume shown in Figure S2 is given by

$$W_P = P\Delta V \tag{2}$$

where P is the input air pressure, ΔV is the increase of volume calculated by

$$\begin{aligned}\Delta V &= V_{bent} - V_{init} \\ &= \left(\int_0^r 2\sqrt{r^2 - z^2}\left(L + \theta\left(\frac{t}{2} + a + b + z\right)\right)dz + \int_0^b e\left(L + \theta\left(\frac{t}{2} + a + z\right)\right)dz\right) - \left(\frac{\pi r^2 L}{2} + ebL\right) \\ &= \left(\int_0^r 2\sqrt{r^2 - z^2}\left(\frac{t}{2} + a + b + z\right)dz + \int_0^b e\left(\frac{t}{2} + a + z\right)dz\right)\theta \\ &= \left(\frac{\pi}{2}\left(\frac{t}{2} + a + b\right)r^2 + eb\left(\frac{t}{2} + a\right) + \frac{eb^2}{2} + \frac{2r^3}{3}\right)\theta\end{aligned} \tag{3}$$

where a is the wall thickness, b is the internal rectangular height, e is the internal chamber width, r is the internal circular radius, t is the thickness of torque compensating layer, L is the internal chamber length (L_p for PIP segment, L_m for MCP segment), $N.A.$ is the neutral axis, P is the input pressure, dz is the differential height element.

Bending strain energy in the 3D printed actuator body W_A. An incompressible Ogden 2-Parameter model is selected to model the adopted 3D printed silicone rubber for Soft-Elastic Composite Actuator. The complete strain energy density function is given by

$$w_m = \sum_{n=1}^{2} \frac{\mu_n}{\alpha_n}\left(\lambda_1^{\alpha_n} + \lambda_2^{\alpha_n} + \lambda_3^{\alpha_n} - 3\right) \tag{4}$$

where λ_1, λ_2 and λ_3 are stretches in axial, circumferential and radial direction. As adopted in [2] and [4], principle stretch λ is defined for axial stretch λ_1, circumferential stretch remains unchanged ($\lambda_2 = 1$), and radial stretch λ_3 is defined as λ^{-1}. To substitute them into equation (4), we can simplify the function to be

$$w_m = \sum_{n=1}^{2} \frac{\mu_n}{\alpha_n}\left(\lambda^{\alpha_n} + \lambda^{-\alpha_n} - 2\right) \tag{5}$$

In the bended state, the stored bending strain energy W_A is distributed to the side walls W_{side}, bottom layer W_{bottom} and hemi-cylinder top layer W_{top} of the 3D printed actuator body. To integrate the strain energy density function over the undeformed volume of different parts (volume remains unchanged due to incompressibility of silicone rubber), the strain energy is given by

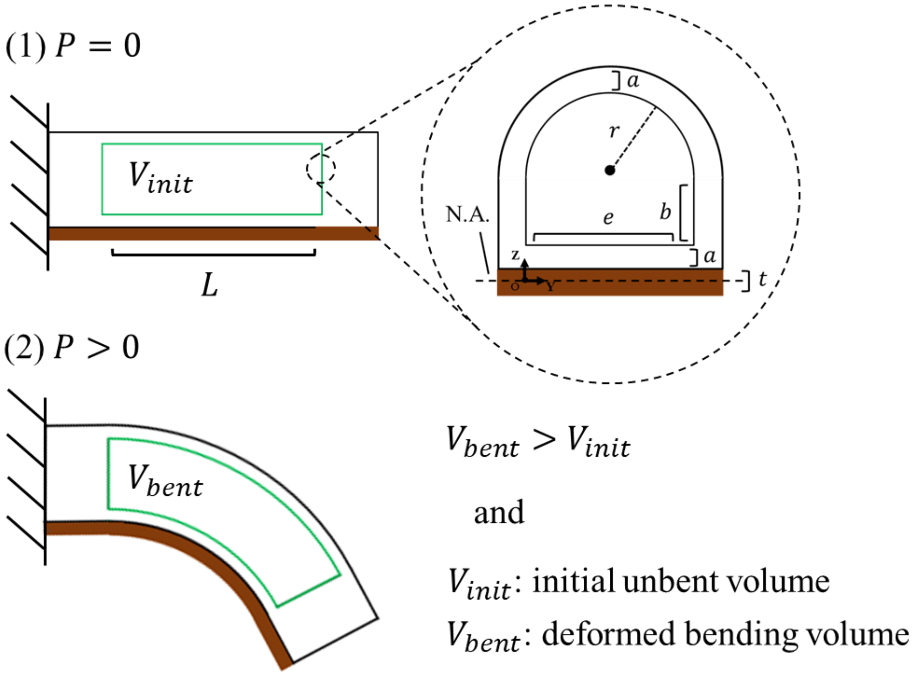

Fig. S2. Volume change of the internal chamber during bending. Cross-sectional area of the Soft-Elastic Composite Actuator for the calculation of deformed volume.

$$W_A = W_{top} + W_{side} + W_{bottom} \tag{6}$$

that

$$\begin{aligned} W_{top} &= \int_0^a \int_0^{\frac{\pi}{2}} 2w_m L\left(\frac{t}{2} + a + b + (r+\tau)\sin\varphi\right) d\varphi d\tau \\ W_{side} &= \int_0^b 2w_m aL \cdot dz \\ W_{bottom} &= \int_0^a 2w_m (r+a)L \cdot dz \end{aligned} \tag{7}$$

Besides, the principle stretches λ_T at hemi-cylinder top layer and λ_S, λ_B at side walls and bottom layer are

$$\begin{aligned} \lambda_T &= \frac{\theta}{L}\left(\frac{t}{2} + a + b + (r+\tau)\sin\varphi\right) + 1 \\ \lambda_S &= \lambda_B = \frac{\theta z}{L} + 1 \end{aligned} \tag{8}$$

Approximation. It is trivial that the integrals listed in equation (7) cannot be easily solved analytically. Solving the bending strain energy stored in the 3D printed silicone rubber must only require numerical integration, which restricts applying the model for further analyzing the bending behavior of Soft-Elastic Composite Actuator. Therefore, we simplify the bending strain energy by i) replacing the hemi-cylinder top layer with an equivalent zero thickness flat rectangular top layer with identical volume at a distance of $h = (a + b) + v(r + a)$ from the side walls, as already presented in [5], and ii) ignoring t (thickness of torque compensating layer), W_{side} and W_{bottom}, due to their negligible contribution. With this approximation, principle stretch λ_T is simplified as

$$\lambda_T = \frac{L + \theta h}{L} \tag{9}$$

and substitute it into the hemi-cylinder top layer strain energy W_{top} in equation (6) and (7), the bending strain energy W_A becomes

$$\begin{aligned} W_A \approx W_{Top} &\approx \int_0^a \int_0^{\frac{\pi}{2}} 2 w_m L\left(a + b + (r+\tau)\sin\varphi\right) d\varphi d\tau \\ &\approx w_m \cdot aL\left(a + \pi\left(a+b\right) + 2r\right) \end{aligned} \tag{10}$$

that

$$w_m = \frac{\mu_1\left(\lambda_T{}^{\alpha_1} - 1\right)^2}{\alpha_1 \lambda_T{}^{\alpha_1}} + \frac{\mu_2\left(\lambda_T{}^{\alpha_2} - 1\right)^2}{\alpha_2 \lambda_T{}^{\alpha_2}} \tag{11}$$

and the parameter v is determined by comparing the exact solution of W_{top} in equation (6) with the approximated expression of W_{top} in equation (10), which $v = 0.655$ can lead to only maximum 8% of difference between them for MCP and PIP segments in the bending range of 0 to 90 degrees. To consider all the simplification in the bending strain energy W_A, the approximated value in equation (10) yields less than 10% of error to the full solution in equation (6), meaning that the simplification is acceptable for further analysis.

Bending strain energy in the torque compensating layer W_L. To consider the bending of torque compensating layer, it can be modeled as a cantilever beam subjected to a pure bending moment

[6]. In that case, the total strain energy stored in the bending torque compensating layer is simply defined by

$$W_L = \frac{M^2 L}{2EI} = \frac{EI}{2L}\theta^2 \tag{12}$$

where M is the bending moment on the torque compensating layer calculated by

$$M = \frac{\theta EI}{L} \tag{13}$$

and EI is the flexural rigidity (E is the Young's modulus and I is the second moment of area) of the torque compensating layer (A2 stainless steel plate).

From the sum of energy, as in equation (1), we can eventually obtain an analytical expression for the bending angle under different input pressure applied in the chambers, which is

$$P = f_1(\theta) + aL\left(a + \pi\left(a+b\right) + 2r\right) \cdot f_2(\theta) \tag{14}$$

that

$$f_1(\theta) = \frac{\theta EI}{2L\left(\frac{\pi}{2}(a+b)r^2 + eba + \frac{eb^2}{2} + \frac{2r^3}{3}\right)}$$
$$f_2(\theta) = \frac{\mu_1\alpha_2\lambda_T{}^{\alpha_2}\left(\lambda_T{}^{\alpha_1}-1\right)^2 + \mu_2\alpha_1\lambda_T{}^{\alpha_1}\left(\lambda_T{}^{\alpha_2}-1\right)^2}{\alpha_1\alpha_2\lambda_T{}^{\alpha_1}\lambda_T{}^{\alpha_2}\left(\frac{\pi}{2}\left(a+b\right)r^2 + eba + \frac{eb^2}{2} + \frac{2r^3}{3}\right)\theta} \tag{15}$$

S1.2.2 Constrained Bending on Model Fingers

To estimate the finger stiffness due to poststroke spasticity using the 3D printed Soft-Elastic Composite Actuator, we need to consider the quasi-static bending on finger joints. To introduce

the work done of the joints at different angular position, the conversation of energy on the 3D printed Soft-Elastic Composite Actuator is

$$W_P = \begin{cases} W_A + W_L - W_{Joint}, & \theta \in \left[0,\ \theta_0\right) \\ W_A + W_L, & \theta = \theta_0 \\ W_A + W_L + W_{Joint}, & \theta \in \left(\theta_0,\ 90°\right] \end{cases} \tag{16}$$

where θ_0 is the resting angle that no work is done on the joints and always less than 90º, W_{Joint} is the elastic potential energy (work done) of the finger joints. Since the dynamics associated with the fingers and the Soft-Elastic Composite Actuator are excluded in the quasi-static model, energy stored in the joints at different position is given by

$$W_{Joint} = \frac{1}{2}k(\theta - \theta_0)^2 \tag{17}$$

where k is the joint stiffness. To treat the finger joint angle to be the same as the bending angle of 3D printed Soft-Elastic Composite Actuator, the pressure-angle relationship of 3D printed Soft-Elastic Composite Actuator on the fingers is dependent on the conditions that

(1) $\theta \geq 0$ and $\theta < \theta_0$

$$P = \frac{2w_m aL^2\left(a + \pi\left(a+b\right) + 2r\right) + EI\theta^2 - kL\left(\theta - \theta_0\right)^2}{2L\left(\frac{\pi}{2}\left(\frac{t}{2} + a + b\right)r^2 + eb\left(\frac{t}{2} + a\right) + \frac{eb^2}{2} + \frac{2r^3}{3}\right)\theta} \tag{18}$$

(2) $\theta = \theta_0$

$$P = \frac{2w_m aL^2\left(a + \pi\left(a+b\right) + 2r\right) + EI\theta^2}{2L\left(\frac{\pi}{2}\left(\frac{t}{2} + a + b\right)r^2 + eb\left(\frac{t}{2} + a\right) + \frac{eb^2}{2} + \frac{2r^3}{3}\right)\theta} \tag{19}$$

(3) $\theta > \theta_0$ and $\theta \leq 90°$

$$P = \frac{2w_m aL^2\left(a + \pi\left(a+b\right) + 2r\right) + EI\theta^2 + kL\left(\theta - \theta_0\right)^2}{2L\left(\frac{\pi}{2}\left(\frac{t}{2} + a + b\right)r^2 + eb\left(\frac{t}{2} + a\right) + \frac{eb^2}{2} + \frac{2r^3}{3}\right)\theta} \tag{20}$$

S1.2.3 Joint Stiffness Estimation

It is more often in real situations that finger joint stiffness is an unknown variable, e.g. the stiff fingers on stroke patients. Here, we can predict the finger joint stiffness based on the individual measured MCP and PIP joint angles at different input pressures. To accomplish this, we need to rearrange equation (18) to (20) to

(1) $\theta \geq 0$ and $\theta < \theta_0$

$$k = \frac{2w_m aL^2\left(a+\pi\left(a+b\right)+2r\right)+EI\theta^2-2PL\left(\frac{\pi}{2}\left(\frac{t}{2}+a+b\right)r^2+eb\left(\frac{t}{2}+a\right)+\frac{eb^2}{2}+\frac{2r^3}{3}\right)\theta}{L\left(\theta-\theta_0\right)^2} \quad (21)$$

(2) $\theta > \theta_0$ and $\theta \leq 90°$

$$k = \frac{2PL\left(\frac{\pi}{2}\left(\frac{t}{2}+a+b\right)r^2+eb\left(\frac{t}{2}+a\right)+\frac{eb^2}{2}+\frac{2r^3}{3}\right)\theta-2w_m aL^2\left(a+\pi\left(a+b\right)+2r\right)-EI\theta^2}{L\left(\theta-\theta_0\right)^2} \quad (22)$$

As mentioned in the manuscript, because only the joint stiffness upon extending the fingers is of our interest, further flexion of the fingers after resting angle (i.e. $\theta \in (\theta_0, 90°]$) would not be considered into stiffness estimation. However, singularity occurs to the mathematical model when the bending angle is equal to the unstretched angle of the flexed joints (i.e. $\theta = \theta_0$), meaning that stiffness estimation cannot be performed only at the point which only small difference between the actuator and joint resting angles. Therefore, it is crucial to define the possible ranges of MCP and PIP joint angles and input pressures for the joint stiffness equation (Eq. 21) as

$$\theta_m \in \left[0,\ \gamma\theta_{0\_m}\right] \text{ and } \theta_p \in \left[0,\ \gamma\theta_{0\_p}\right],\ \ 0<\gamma<1 \quad (23)$$

$$P = \text{cutoff} \ \text{ when } \left(\theta_m > \gamma\theta_{0\_m} \text{ or } \theta_p > \gamma\theta_{0\_p}\right) \quad (24)$$

where γ is an empirical coefficient chosen to be 0.7 to avoid reaching singularity (refer to the following section). In the bending state of the 3D printed Soft-Elastic Composite Actuator, cutoff pressure is defined as soon as the measured MCP or PIP joint angle exceeds its upper limit ($\gamma\theta_{0\_m}$ or $\gamma\theta_{0\_p}$), and therefore the Soft-Elastic Composite Actuator would not be further actuated and influenced by the singularity in the model.

S2 Experimental Validation

In this section, we will validate the mathematical models experimentally. We will test the bending performance of Soft-Elastic Composite Actuator in free space and on model fingers (**see Video S1**). An instruction is provided as a guidance of wearing the Soft-Elastic Composite Actuator.

S2.1 Experimental Setup

The architecture of the pneumatic control setup for bending angle measurement is shown in Figure S3. The air pump (BTC Diaphragm Pump, Parker Hannifin Corporation, Ohio, U.S.A.) supplies air pressure for the Soft-Elastic Composite Actuator via a pressure meter (ZSE20C(F), SMC Pneumatic, Tokyo, Japan) and a pressure regulator (IR2020-02BG, SMC Pneumatic, Tokyo, Japan). The pressure regulator can be manually adjusted to control the supplied air pressure to the Soft-Elastic Composite Actuator. The pressure value is displayed on the screen of the pressure sensor. A 12V voltage source is also connected to the pressure sensor and air pump for operation.

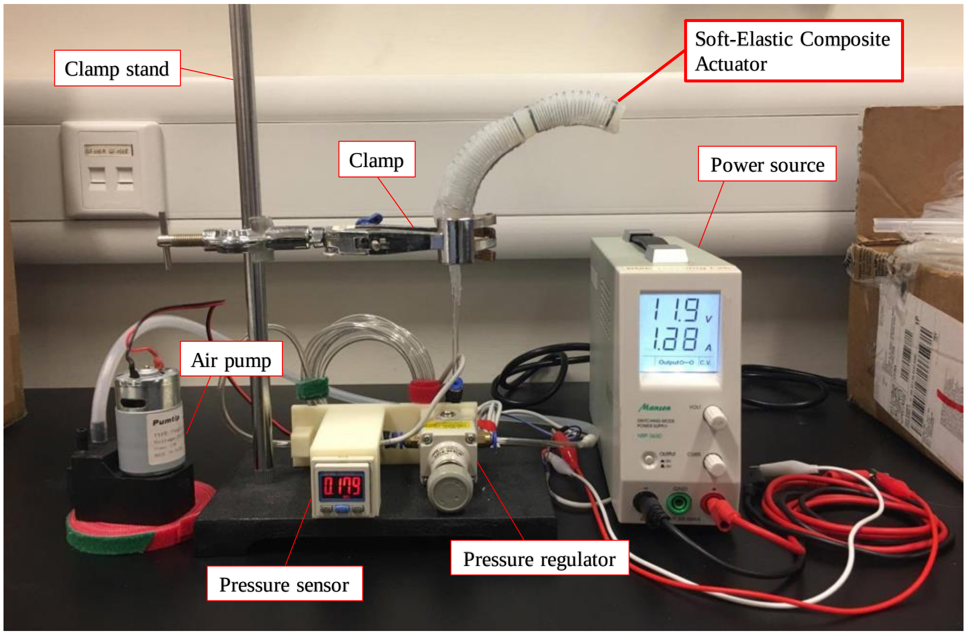

Fig. S3. Example experimental setup in free space bending measurement. A power source is connected to provide voltage for the air pump and pressure sensor. The pressure regulator is set to adjust the air pressure for the Soft-Elastic Composite Actuator. A clamp stand is used to fix the actuator into a specific position

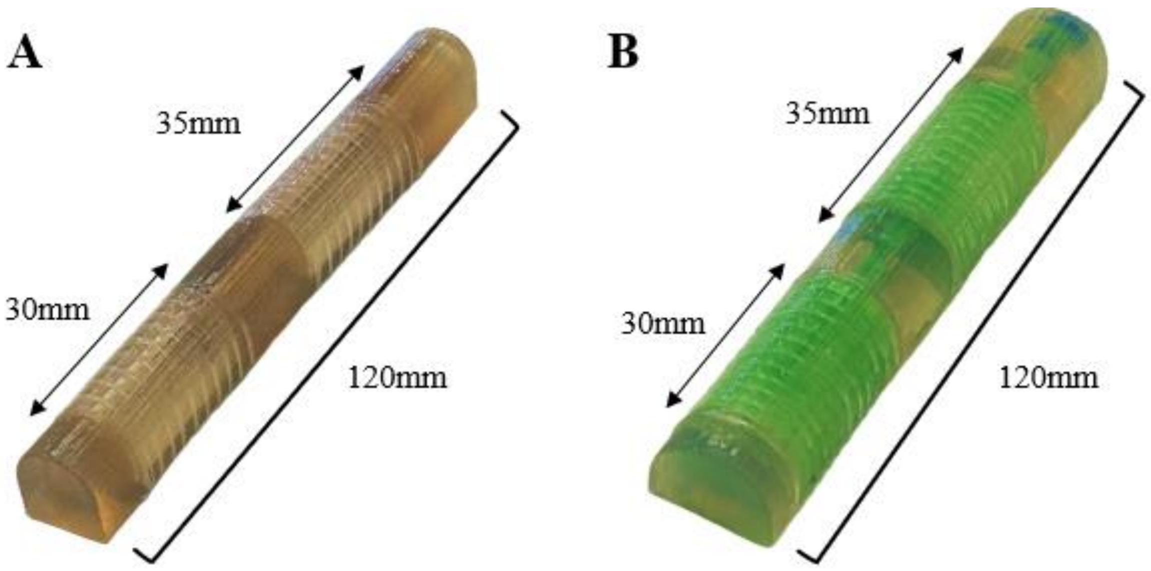

Fig. S4. 3D printed prototype of the baseline *(A)* semi-obround and *(B)* semi-circular Soft-Elastic Composite Actuator.

Same settings apply to both free space bending measurement and constrained bending on model fingers, which the bending angle of the 3D printed Soft-Elastic Composite Actuator is captured and measured using the image analyzing software (ImageJ, National Institute of Health, Maryland, U.S.A.).

Two baseline sets of 3D printed Soft-Elastic Composite Actuators are designed for all the following experiments, named the semi-obround Soft-Elastic Composite Actuator and semi-circular Soft-Elastic Composite Actuator. Additional two variations are also applied to the thickness of torque compensating layers (0.1mm, 0mm that represents the strain limiting layer). Figure S4 shows the real prototypes of baseline semi-obround and semi-circular Soft-Elastic Composite Actuator.

S2.2 Free Space Bending Measurement

Pressures of 0 kPa, 40 kPa, 80 kPa, 120 kPa and 160 kPa are applied to the semi-obround and semi-circular Soft-Elastic Composite Actuator respectively. The bending angle at different input pressure is measured accordingly. Six actuators, which three semi-obround Soft-Elastic Composite

Table I. Semi-Obround Soft-Elastic Composite Actuator

Baseline actuator (0.2mm thickness of torque compensating layer)

Pressure (kPa)	FEM	Analytical	Experiment
0	0º	0º	2º
40	21.8º	16.6º	16º
80	39.0º	34.1º	29º
120	56.5º	53.5º	51º
160	71.7º	76.1º	77º

Variation 1 (0.1mm thickness of torque compensating layer)

Pressure (kPa)	FEM	Analytical	Experiment
0	0º	0º	1º
40	24.8º	19.5º	19º
80	47.5º	42.4º	37º
120	67.1º	66.9º	63º
160	87.0º	94.5º	101º

Variation 2 (no torque compensating layer, using traditional strain-limiting layer)

Pressure (kPa)	FEM	Analytical	Experiment
0	0º	0º	2º
40	21.4º	20.2º	19º
80	46.6º	43.4º	37º
120	75.0º	69.2º	63º
160	106.3º	98.2º	101º

Table II. Semi-Circular Soft-Elastic Composite Actuator

Baseline actuator (0.2mm thickness of torque compensating layer)

Pressure (kPa)	FEM	Analytical	Experiment
0	0°	0°	2°
40	25.7°	26.3°	19°
80	56.9°	56.1°	45°
120	94.5°	88.1°	75°
160	136.3°	124.0°	116°

Variation 1 (0.1mm thickness of torque compensating layer)

Pressure (kPa)	FEM	Analytical	Experiment
0	0°	0°	1°
40	29.6°	34.0°	25°
80	76.3°	74.1°	66°
120	124.8°	120.9°	132°
160	172.5°	177.7°	192°

Variation 2 (no torque compensating layer, using traditional strain-limiting layer)

Pressure (kPa)	FEM	Analytical	Experiment
0	0°	0°	0°
40	35.2°	35.7°	22°
80	81.4°	78.4°	66°
120	127.1°	129.7°	132°
160	187.6°	190.2°	192°

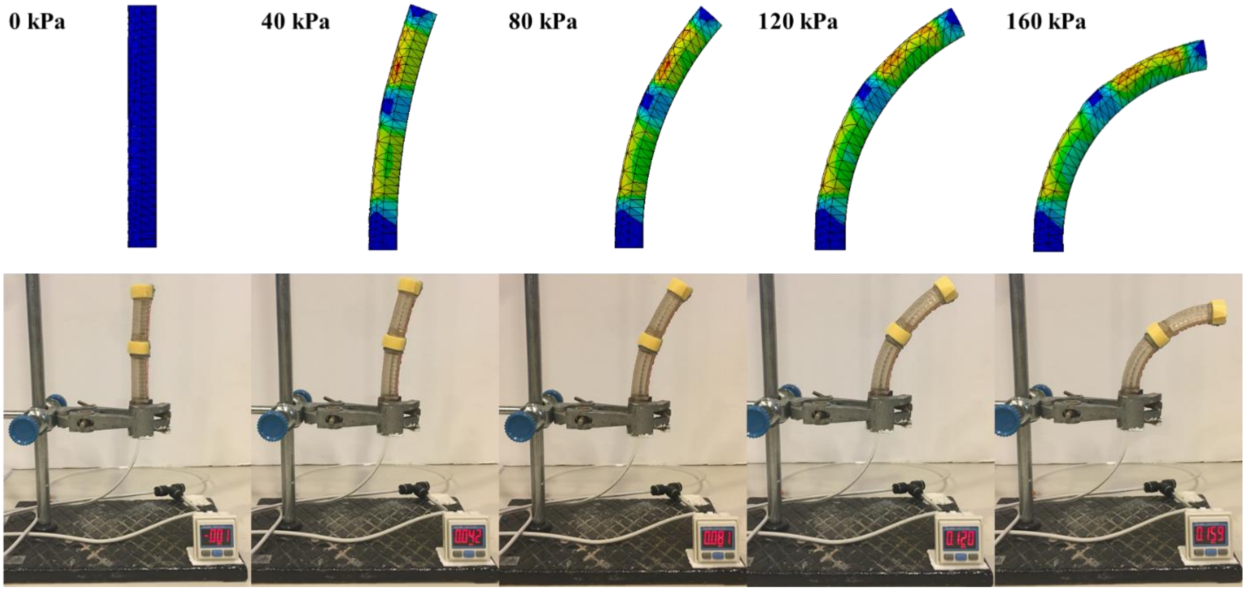

Fig. S5. Bending of the **baseline semi-obround** Soft-Elastic Composite Actuator at the input pressure of 0 kPa, 40 kPa, 80 kPa, 120 kPa and 160 kPa. Upper section is the finite element simulation results, and lower section is the experimental results.

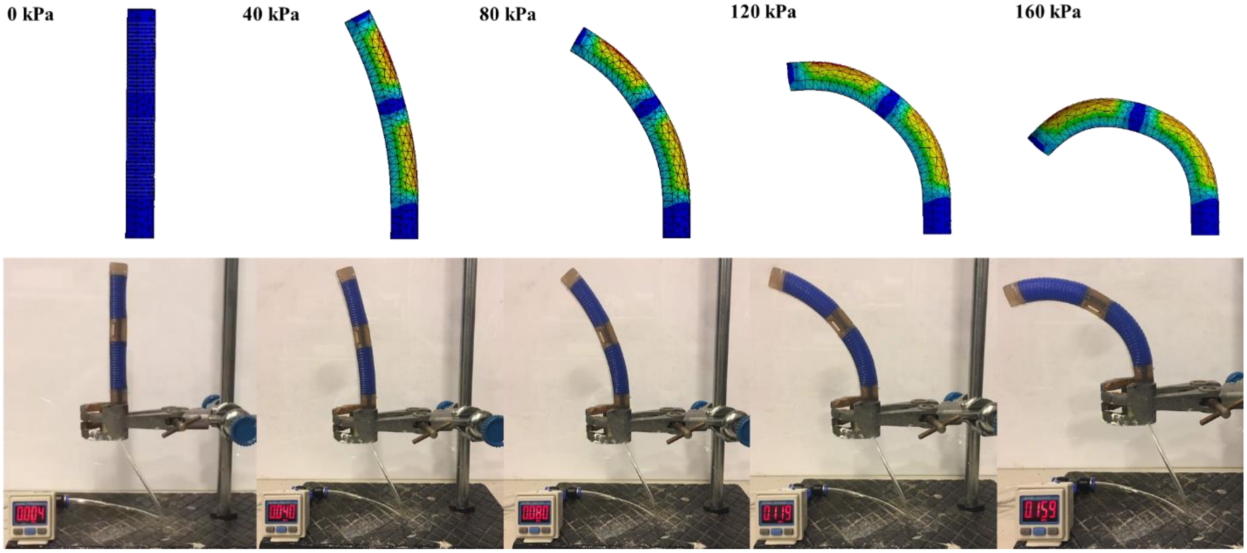

Fig. S6. Bending of the **baseline semi-circular** Soft-Elastic Composite Actuator at the input pressure of 0 kPa, 40 kPa, 80 kPa, 120 kPa and 160 kPa. Upper section is the finite element simulation results, and lower section is the experimental results.

Actuators (thickness of torque compensating layer to be 0.2mm (baseline), 0.1mm, 0mm as fiber-reinforced actuator) and three semi-circular Soft-Elastic Composite Actuators (thickness of torque compensating layer to be 0.2mm (baseline), 0.1mm, 0mm as fiber-reinforced actuator), are tested to verify the analytical model (Eq. 14 to 15) and the finite element simulation.

Table I and II list the bending angles of all six actuators at different input pressures in details. From the results, we can see the bending behavior of the 3D printed Soft-Elastic Composite Actuator (for example, Figure S5 and S6) can be captured by both the analytical model and the finite element simulation. This can aid further results of prediction and optimization before 3D printing.

S2.3 Constrained Bending Measurement on Model Fingers

Since the 3D printed Soft-Elastic Composite Actuator is attached to a hand with Velcro straps, the overall results of constrained bending on model fingers would vary with each use if there is not a standardized procedure of wearing the Soft-Elastic Composite Actuator. To address consistency in wearing of the actuator, particularly for the human subjects, an instruction is provided prior to the experiments. Performance of the Soft-Elastic Composite Actuator on model fingers are then concluded in terms of the bending angles on fingers and the accuracy of joint stiffness estimation using the actuator.

S2.3.1 Actuator Installation

When wearing the Soft-Elastic Composite Actuator, the MCP and PIP segments of the actuator are aligned with the MCP and PIP joints of the human finger respectively. Once aligned, the proximal end (the end connected with air tube) of the actuator would be positioned on the base of the hand. Then, Velcro straps are used to secure each finger on the Soft-Elastic Composite Actuator to minimize any gap between the finger and the actuator.

Figure S7 illustrates proper wearing of the Soft-Elastic Composite Actuator to a hand. The midpoints of MCP and PIP segments are aligned to the center of MCP and PIP joints. For human

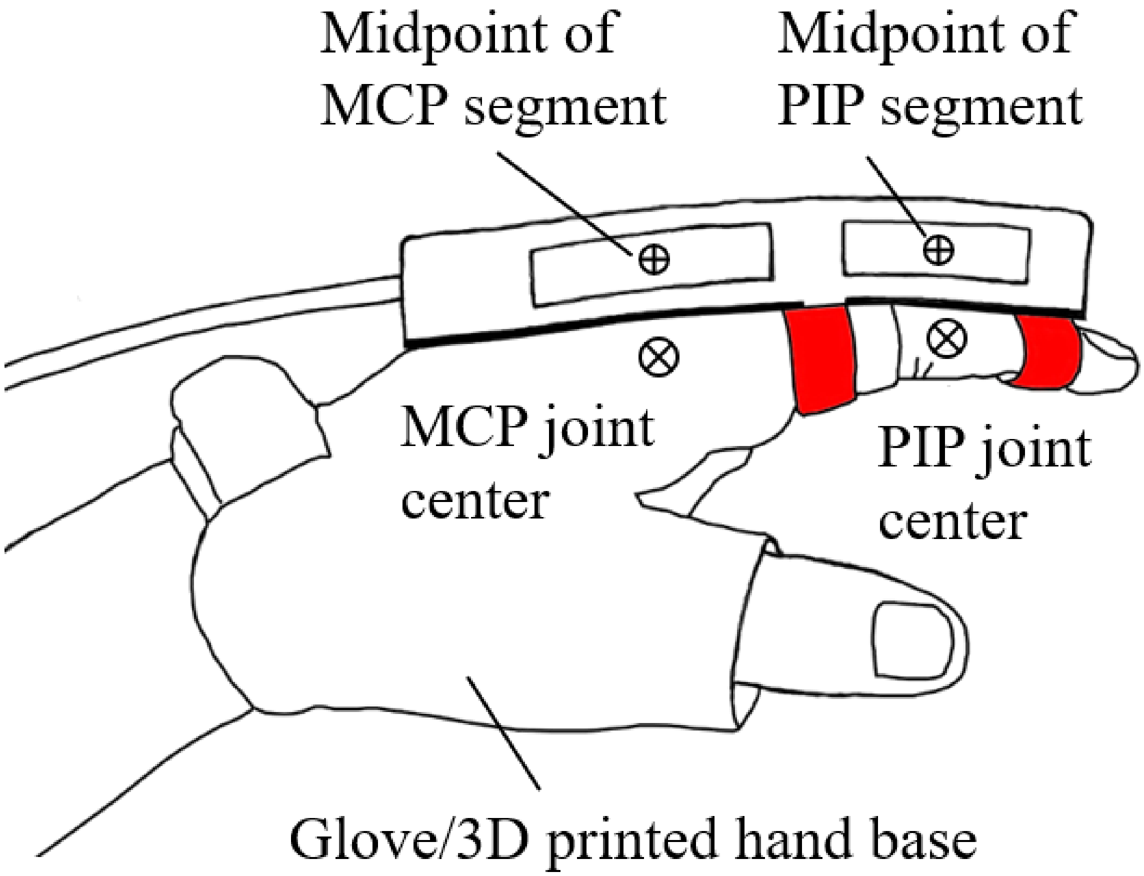

Fig. S7. Proper wearing of 3D printed Soft-Elastic Composite Actuator onto a hand

subjects, the proximal end of the actuator is fixed onto a glove or a 3D printed hand base, forming a soft robotic hand that enables the 3D printed Soft-Elastic Composite Actuator to be fully secured to a hand.

S2.3.2 Bending Angles on model fingers

Two mannequin hands in which the index fingers are installed with torsion springs at the PIP and MCP joint positions are designed as low and high stiffness fingers. Design rationales of the model index fingers have been addressed in our previous work [2]. Baseline semi-obround and semi-circular Soft-Elastic Composite Actuator are utilized. Pressures of 0 kPa, 20 kPa, 40 kPa, 60 kPa, 80 kPa, 100 kPa, 120 kPa, 140 kPa and 160 kPa are applied to two actuators respectively. Table III and IV list the bending angles of the two actuators under different input pressures (for example,

Table III. Low Stiffness Finger

Baseline semi-obround Soft-Elastic Composite Actuator

Pressure (kPa)	Analytical		Experiment	
0	47.0º		46º	
20	49.7º		47º	
40	52.7º		50º	
60	56.1º		54º	
80	59.9º	ROM = 37.2º	59º	ROM = 41º
100	64.8º		65º	
120	69.9º		71º	
140	76.2º		78º	
160	84.2º		87º	

Baseline semi-circular Soft-Elastic Composite Actuator

Pressure (kPa)	Analytical		Experiment	
0	47.2º		47º	
20	51.4º		48º	
40	56.3º		55º	
60	62.7º		62º	
80	70.5º	ROM = 70.8º	71º	ROM = 65º
100	80.7º		81º	
120	92.6º		91º	
140	104.7º		102º	
160	118.0º		112º	

Table IV. High Stiffness Finger

Baseline semi-obround Soft-Elastic Composite Actuator

Pressure (kPa)	Analytical		Experiment	
0	86.9º		85º	
20	89.0º		87º	
40	91.1º		89º	
60	93.4º		91º	
80	95.9º	ROM = 21.4º	96º	ROM = 32º
100	98.6º		101º	
120	101.5º		106º	
140	104.7º		112º	
160	108.3º		117º	

Baseline semi-circular Soft-Elastic Composite Actuator

Pressure (kPa)	Analytical		Experiment	
0	87.0º		88º	
20	90.1º		90º	
40	93.5º		94º	
60	97.4º		98º	
80	101.7º	ROM = 42.2º	101º	ROM = 42º
100	106.7º		105º	
120	112.5º		112º	
140	119.6º		121º	
160	129.2º		130º	

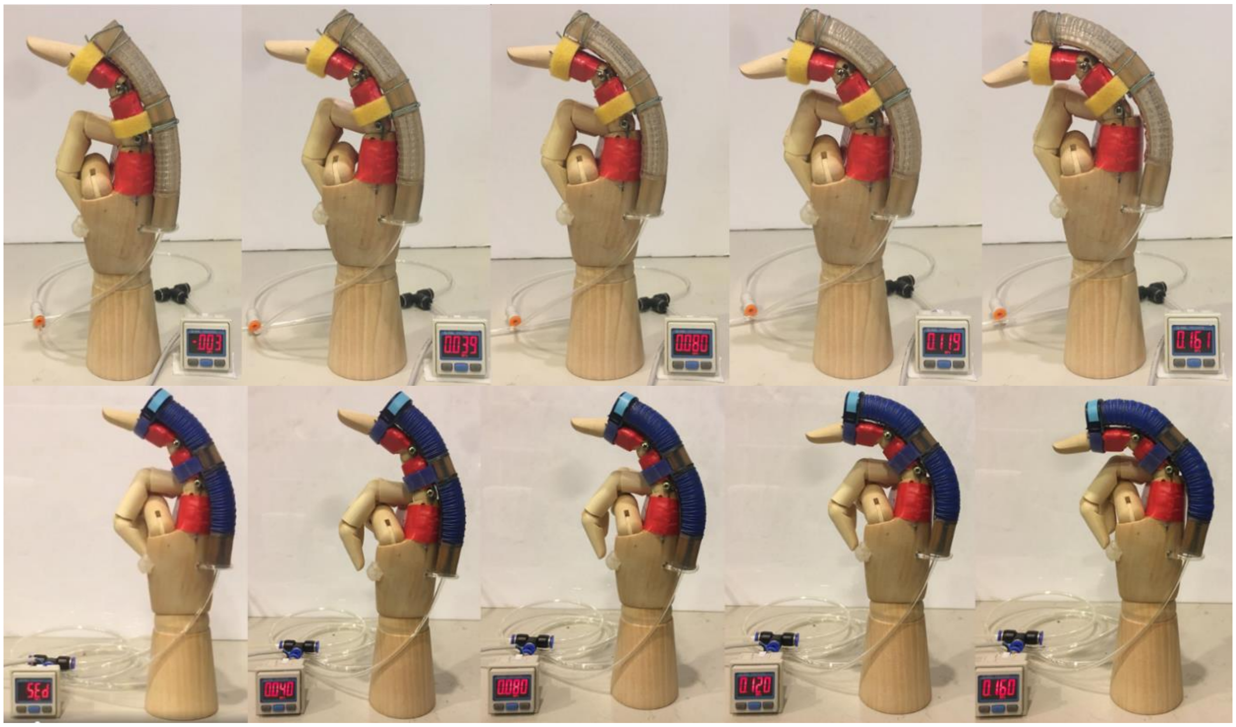

Fig. S8. Flexion and Extension of the **Low Stiffness Finger**. Upper section is the **baseline semi-obround** actuator, and lower section is the **baseline semi-circular** actuator.

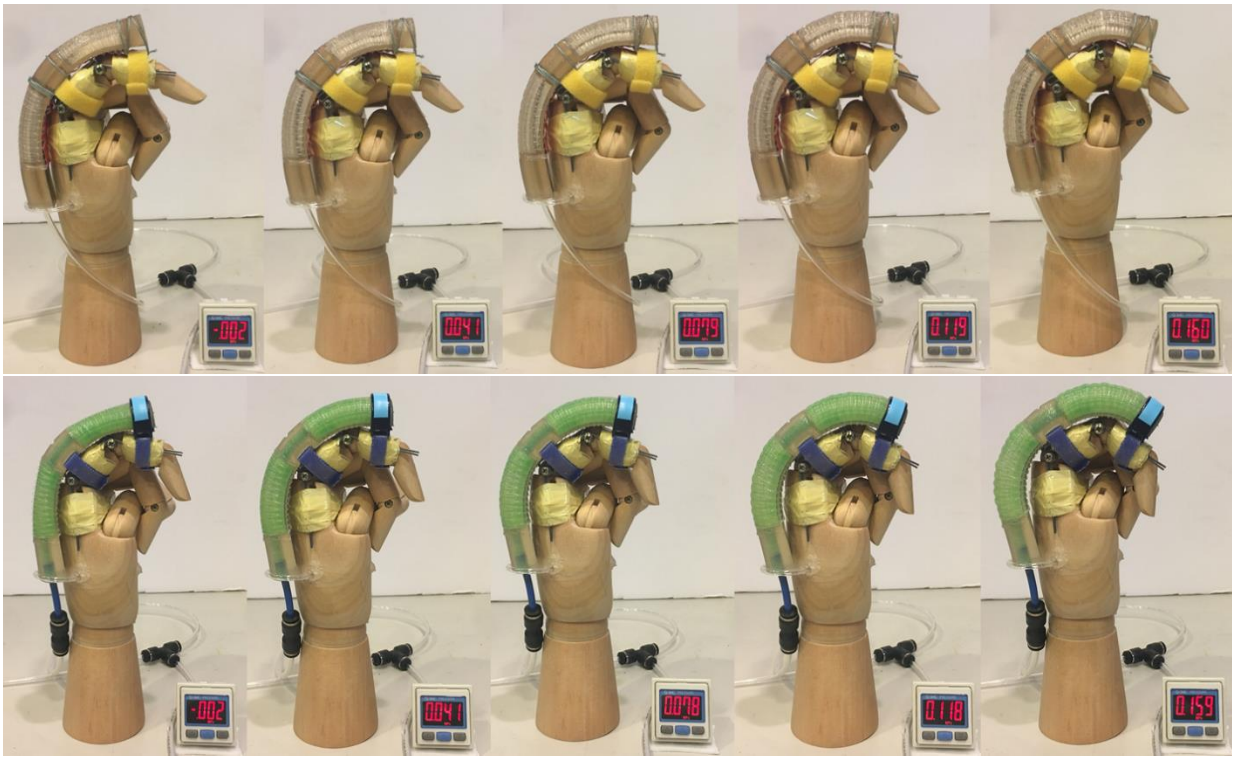

Fig. S9. Flexion and Extension of the **High Stiffness Finger**. Upper section is the **baseline semi-obround** actuator, and lower section is the **baseline semi-circular** actuator.

Figure S8 and S9) in details. Satisfactory match between the model and experimental results is observed. For a single actuator, the facilitated extension of Low Stiffness Finger is larger than that of High Stiffness Finger. The range-of-motion (ROM) of High Stiffness Finger is also less than that of Low Stiffness Finger, while a larger ROM can be provided to the fingers if baseline semi-circular Soft-Elastic Composite Actuator is adopted.

S2.3.3 Stiffness Estimation of the model fingers

It is obvious that the stiffness of impaired fingers on stroke patients tends to be unknown. On the model index fingers, i.e. Figure S8 and S9, we adopt equation (21) to demonstrate stiffness estimation based on the individual measured MCP and PIP joint angles at different input pressures.

We presume that there is no preliminary information of the characteristics of the fingers. First, we record the bending angle of the 3D printed Soft-Elastic Composite Actuator and the corresponding pressure in the bending state. Then, we substitute the collected data to equation (20) for the calculation of joint stiffness values. The estimated stiffness values are compared with the original values we designed for the model index fingers for accuracy validation.

Table V and VI conclude the estimated stiffness of MCP and PIP joints on both High and Low Stiffness Finger. It is worth to note that reasonable results cannot be guaranteed when the bending angle of each segment further increases with the input pressure (e.g. negative values for joint stiffness). In equation (21), singularity occurs when the segment angle is equal to the initial resting angle of the joint. From our experience, near the singularity point, i.e. when the difference between two angle values is small, accuracy of the estimated stiffness would be influenced significantly. To avoid it, there should be a defined upper limit of the segment angle ($\gamma\theta_{0\_m/p}$) such that end of the actuation of the 3D printed Soft-Elastic Composite Actuator is indicated when the angle has exceeded the limit (Eq. 23 and 24). $\gamma = 0.7$ is empirically determined to be a suitable coefficient that can exclude the unreliable results but retain most of the reasonable results. Eventually, the stiffness of the joint is taken by the average of the retained results.

Table V. Estimated stiffness of the model MCP and PIP joints

(with Semi-Obround Soft-Elastic Composite Actuator, γ = 0.7)

**Low Stiffness Finger**						**High Stiffness Finger**					
PIP joint<br>Characteristic:<br>$k_p = 0.3372$ Nm/rad<br>$\theta_{0\_p} = 60^o$, $\gamma\theta_{0\_p} = 42^o$			MCP joint<br>Characteristic:<br>$k_p = 0.1476$ Nm/rad<br>$\theta_{0\_m} = 40^o$, $\gamma\theta_{0\_m} = 28^o$			PIP joint<br>Characteristic:<br>$k_p = 0.5508$ Nm/rad<br>$\theta_{0\_p} = 75^o$, $\gamma\theta_{0\_p} = 52.5^o$			MCP joint<br>Characteristic:<br>$k_p = 0.7387$ Nm/rad<br>$\theta_{0\_m} = 70^o$, $\gamma\theta_{0\_m} = 49^o$		
P (KPA)	θ_p	k_p (NM/RAD)	P (KPA)	θ_m	k_m (NM/RAD)	P (KPA)	θ_p	k_p (NM/RAD)	P (KPA)	θ_m	k_m (NM/RAD)
0	30º	0.3174	0	16º	0.13	0	42º	0.4909	0	43º	0.6717
20	30º	0.2722	20	17º	0.0923	20	43º	0.4883	20	44º	0.6676
40	31º	0.2612	40	19º	0.1196	40	44º	0.4819	40	45º	0.657
60	33º	0.2842	60	21º	0.1134	60	45º	0.4711	60	46º	0.6383
80	35º	0.3059	80	24º	0.1286	80	48º	0.5804	80	48º	0.7022
100	37º	0.3245	100	28º	0.2	-	-	-	-	-	-

Table VI. Estimated stiffness of the model MCP and PIP joints

(with Semi-Circular Soft-Elastic Composite Actuator, γ = 0.7)

**Low Stiffness Finger**						**High Stiffness Finger**					
PIP joint<br>Characteristic:<br>$k_p = 0.3372$ Nm/rad<br>$\theta_{0\_p} = 60^o, \gamma\theta_{0\_p} = 42^o$			MCP joint<br>Characteristic:<br>$k_p = 0.1476$ Nm/rad<br>$\theta_{0\_m} = 40^o, \gamma\theta_{0\_m} = 28^o$			PIP joint<br>Characteristic:<br>$k_p = 0.5508$ Nm/rad<br>$\theta_{0\_p} = 75^o, \gamma\theta_{0\_p} = 52.5^o$			MCP joint<br>Characteristic:<br>$k_p = 0.7387$ Nm/rad<br>$\theta_{0\_m} = 70^o, \gamma\theta_{0\_m} = 49^o$		
P (KPA)	θ_p	k_p (NM/RAD)	P (KPA)	θ_m	k_m (NM/RAD)	P (KPA)	θ_p	k_p (NM/RAD)	P (KPA)	θ_m	k_m (NM/RAD)
0	29º	0.2768	0	18º	0.1915	0	45º	0.6738	0	43º	0.6686
20	30º	0.2468	20	18º	0.1153	20	46º	0.6391	20	44º	0.6195
40	33º	0.2802	40	22º	0.1431	40	48º	0.6679	40	46º	0.6329
60	37º	0.3657	60	25º	0.0924	60	49º	0.6051	60	49º	0.7281

S3 Preliminary Evaluation

In this section, we will present the complete platform for finger stiffness measurement on human subjects, and the stiffness values of the MCP and PIP joints measured with the platform. Demographic information of the recruited stroke subjects will also be provided to present their stroke characteristics.

S3.1 Experimental Configuration

The pneumatic control setup for MCP and PIP joint stiffness measurement in shown in Figure S10. For an online evaluation, equation (21) and its conditions (23) and (24) are implemented on

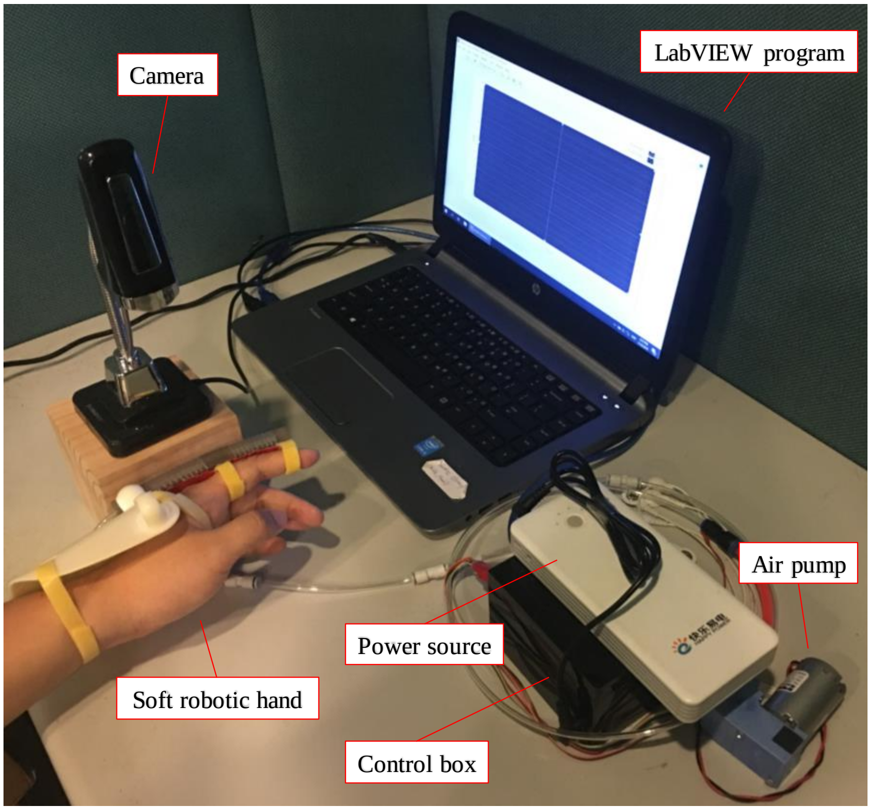

Fig. S10. Example experimental setup in free space bending measurement. The control platform is implemented on a personal computer (64-bit operating system, i5 CPU and 8GB RAM) based on LabVIEW 2017 software. The movement of index finger is assisted by the Soft-Elastic Composite Actuator on the soft robotic hand. The bending motion is captured by the camera and the bending angle is analyzed on PyCharm (Python + OpenCV).

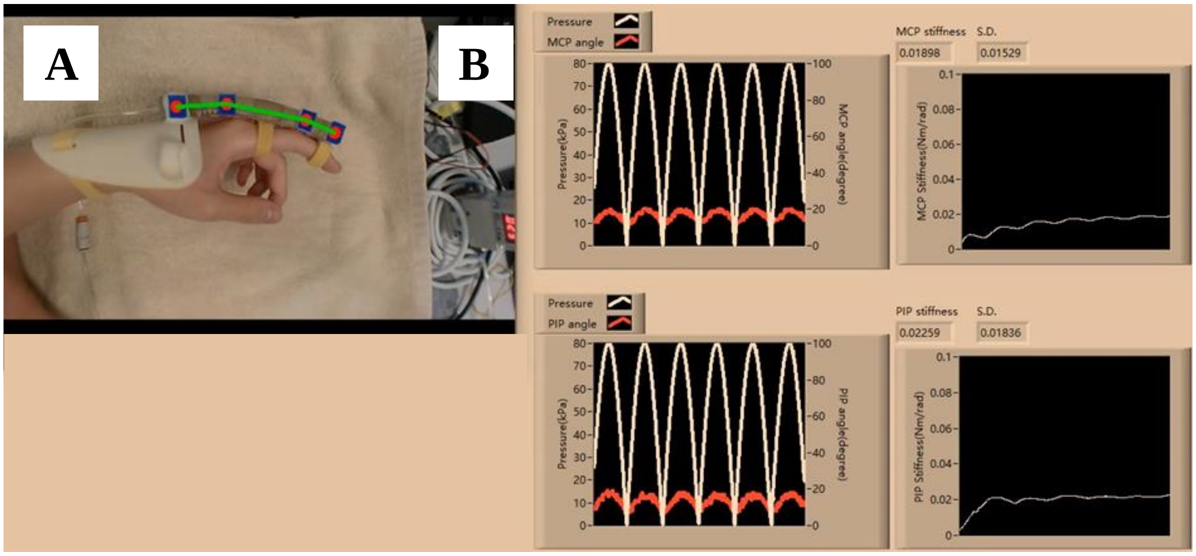

Fig. S11. Interface of the developed LabVIEW program for an online stiffness evaluation. *(A)* Camera capturing the motion of index finger. *(B)* Input pressure and the measure angle of the 3D printed Soft-Elastic Composite Actuator. Joint average stiffness and standard deviation of MCP and PIP joints over time.

LabVIEW 2017 Version (National Instruments Corporation, Texas, U.S.A.) to calculate the stiffness based on the current input pressure and joint angle. The air pump (BTC Diaphragm Pump, Parker Hannifin Corporation, Ohio, U.S.A.) supplies air pressure for the Soft-Elastic Composite Actuator. The control box contains a data acquisition device (DAQ) (USB-6009, National Instruments Corporation, Texas, U.S.A.) and a proportional solenoid valve (ITV2091-21N2BS5, SMC Pneumatics, Tokyo, Japan). The DAQ transmits commands to the valve for regulating the pressure supplied to the 3D printed Soft-Elastic Composite Actuator. Figure S11 shows our control program developed on LabVIEW 2017 that determines the stiffness of MCP and PIP joints by the

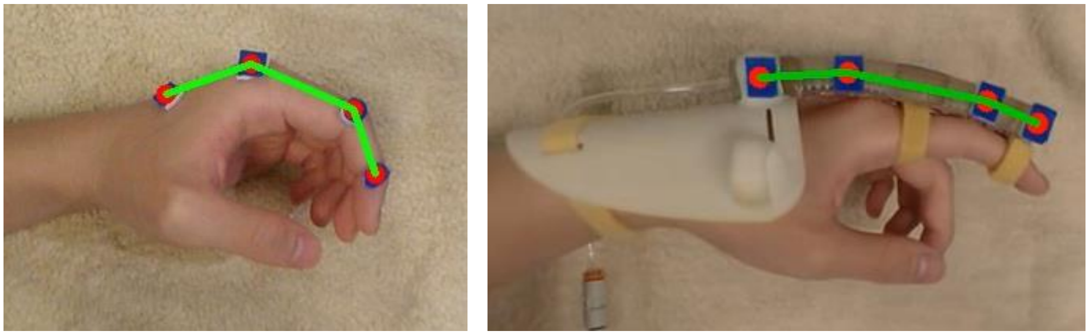

Fig. S12. ***Left:*** Initial flexion of the fingers of a recruited subject upon relaxation. ***Right:*** Finger extension passively facilitated by the 3D printed Soft-Elastic Composite Actuator.

input pressure and the bending angle captured by the camera in Figure S12 (WX150HD720P, Weixin Shijie, Shenzhen, China) and analyzed on PyCharm (Python + OpenCV) (**see Video S2 and S3** for the real application).

To ensure result consistency, the posture of the upper limb needs to be defined during experiments. Figure S13 shows the proper posture of the arm onto the platform. The 3D printed hand base of the soft robotic hand covers the wrist to maintain it in a neutral position (0º of flexion and extension) with respect to the forearm, while the forearm is also being held into its neutral position (0º of supination and pronation). Assistance is provided to laterally fix the position of hand base if the subject fails to accomplish it. The elbow is flexed by 90º and laid on the desk, and no voluntary movement that alter the neutral position of the shoulder should be performed.

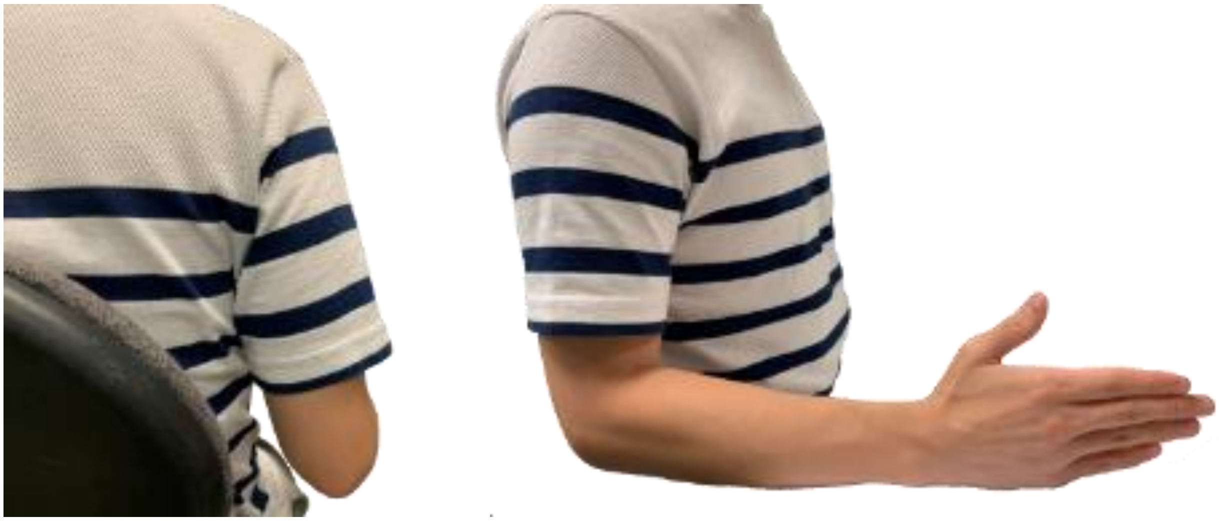

Fig. S13. Defined upper-limb position during the experiments

S3.2 Subjects' demographic information

Four stroke subjects and four healthy subjects are recruited for the preliminary evaluation of their MCP and PIP joint stiffness. It is registered to the Joint Chinese University of Hong Kong-New Territories East Cluster (CUHK-NTEC) Clinical Research Ethics Committee (Ref. ID: NCT03286309). Table VII shows the clinical information of all subjects.

Table VII. Clinical Characteristic

Subject	Age	Gender	Stroke Onset	Stroke Type	Hemiplegic side	ARAT[1]	MAS[2]
**S1**	33	Male	41 months	Hemorrhagic	Left	3	1+
**S2**	58	Female	61 months	Ischemic	Left	7	1+
**S3**	60	Male	22 months	Hemorrhagic	Left	4	3
**S4**	72	Male	34 months	Ischemic	Left	3	1+
**H1**	24	Male	-	-	- (Right)[3]	57	0
**H2**	24	Male	-	-	- (Left)[3]	57	0
**H3**	23	Male	-	-	- (Right)[3]	57	0
**H4**	24	Male	-	-	- (Left)[3]	57	0

[1] ARAT (Action Research Arm Test) is a continuous measurement for the changes in upper limb function among hemiplegic patients with no categorical cutoff scores [7]. Full score is 57. Higher score indicates better upper limb performance [8,9].

[2] Ranging from 0 to 4 (0 = No increase in muscle tone, 1 = Slight increase in muscle tone at the end of the ROM, 1+ = Slight increase in muscle tone throughout less than half of the ROM, 2 = More marked increase in muscle tone throughout most of the ROM, 3 = Considerable increase in muscle tone throughout most of the ROM, 4 = Complete rigid of the affected joint(s)) [10]. Higher MAS score indicates severe spasticity [11]. Flexor tone is assessed for MAS.

[3] Index fingers on the dominant hands are chosen for the assessment.

S3.3 Results of the MCP and PIP joint stiffness

Table VIII. Stiffness results of stroke subjects

	PIP joint					MCP joint				
Subject	P (kPa)	θ_{0_p}	$\gamma\theta_{0\_p}$	θ_p	k_p (Nm/rad)	P (kPa)	θ_{0_m}	$\gamma\theta_{0\_m}$	θ_m	k_m (Nm/rad)
	0			27°	0.1147	0			20°	0.0983
	20			28°	0.0989	20			22°	0.0893
**S1**	40	72°	50.4^{o}	30°	0.0911	40	54°	37.8^{o}	25°	0.0856
	60			33°	0.0898	60			29°	0.0854
	80			35°	0.0675	80			34°	0.0865
Mean					0.09240					0.08902
S.D.					0.01709					0.00542

Subject	P (kPa)	θ_{0_p}	$\gamma\theta_{0\_p}$	θ_p	k_p (Nm/rad)	P (kPa)	θ_{0_m}	$\gamma\theta_{0\_m}$	θ_m	k_m (Nm/rad)
	0			16°	0.0832	0			13°	0.1018
**S2**	20	48°	33.6^{o}	18°	0.0779	20	35°	24.5^{o}	15°	0.0859
	40			21°	0.0793	40			19°	0.0979
	60			26°	0.1166	60			24°	0.1142
Mean					0.08925					0.09995
S.D.					0.01837					0.01166

Subject	P (kPa)	θ_{0_p}	$\gamma\theta_{0\_p}$	θ_p	k_p (Nm/rad)	P (kPa)	θ_{0_m}	$\gamma\theta_{0\_m}$	θ_m	k_m (Nm/rad)
	0			55°	0.7177	0			36°	0.5583
**S3**	20	90°	63°	56°	0.6869	20	61°	42.7^{o}	38°	0.5833
	40			59°	0.7895	40			41°	0.6945
	60			61°	0.8189	60			43°	0.6888
Mean					0.75331					0.63123
S.D.					0.06132					0.07060

Subject	P (kPa)	θ_{0_p}	$\gamma\theta_{0\_p}$	θ_p	k_p (Nm/rad)	P (kPa)	θ_{0_m}	$\gamma\theta_{0\_m}$	θ_m	k_m (Nm/rad)
	0			16°	0.0328	0			16°	0.1673
**S4**	20	67°	46.9^{o}	20°	0.0408	20	37°	25.9^{o}	19°	0.1975
	40			23°	0.0397	40			21°	0.1514
	60			26°	0.0336	60			24°	0.0817
Mean					0.03673					0.14948
S.D.					0.00411					0.04906

Table IX. Stiffness results of healthy subjects

	PIP joint					MCP joint				
Subject	P (kPa)	θ_{0_p}	$\gamma\theta_{0\_p}$	θ_p	k_p (Nm/rad)	P (kPa)	θ_{0_m}	$\gamma\theta_{0\_m}$	θ_m	k_m (Nm/rad)
	0			5°	0.0058	0			6°	0.0067
	20			9°	0.0077	20			11°	0.0106
**H1**	40	44°	30.8^{o}	14°	0.0094	40	46°	32.2^{o}	17°	0.0159
	60			20°	0.014	60			23°	0.0138
	80			26°	0.0098	80			31°	0.0394
Mean					0.00934					0.01728
S.D.					0.00305					0.01284

Subject	P (kPa)	θ_{0_p}	$\gamma\theta_{0\_p}$	θ_p	k_p (Nm/rad)	P (kPa)	θ_{0_m}	$\gamma\theta_{0\_m}$	θ_m	k_m (Nm/rad)
	0			6°	0.0068	0			9°	0.025
**H2**	20	49°	34.3^{o}	9°	0.0059	20	40°	28°	13°	0.031
	40			15°	0.0119	40			16°	0.0143
	60			21°	0.0189	60			24°	0.054
Mean					0.01086					0.03106
S.D.					0.00597					0.01678

Subject	P (kPa)	θ_{0_p}	$\gamma\theta_{0\_p}$	θ_p	k_p (Nm/rad)	P (kPa)	θ_{0_m}	$\gamma\theta_{0\_m}$	θ_m	k_m (Nm/rad)
	0			6°	0.0115	0			11°	0.0161
	20			9°	0.0104	20			14°	0.0146
**H3**	40	39°	27.3^{o}	14°	0.0135	40	58°	40.6^{o}	18°	0.0118
	60			20°	0.0224	60			23°	0.006
	80			26°	0.0187	80			30°	0.0015
Mean					0.01530					0.01000
S.D.					0.00509					0.00612

Subject	P (kPa)	θ_{0_p}	$\gamma\theta_{0\_p}$	θ_p	k_p (Nm/rad)	P (kPa)	θ_{0_m}	$\gamma\theta_{0\_m}$	θ_m	k_m (Nm/rad)
	0			6°	0.0131	0			11°	0.0226
**H4**	20	36°	25.2^{o}	9°	0.0105	20	45°	31.5^{o}	13°	0.0191
	40			15°	0.0203	40			18°	0.0178
	60			22°	0.0594	60			24°	0.0073
Mean					0.02583					0.01670
S.D.					0.02276					0.00659

Semi-circular Soft-Elastic Composite Actuator is adopted for joint stiffness estimation. $\gamma = 0.7$ is used throughout the experiments to define the upper limit of joint angle for all subjects. Table VIII and IX list the detailed results of MCP and PIP joint stiffness upon the measurements.

Values of their MCP and PIP joint stiffness of both stroke and healthy subjects are found to be near the stiffness ranges presented by [12-14] (Stroke: ~0.55 Nm/rad with MAS = 3, Healthy: ~0.03 Nm/rad). Larger joint stiffness is observed on stroke subjects, which the joint stiffness increases with the MAS score. This would be indicative of the condition of the finger, and thus has further potential in clinical application.

Reference

[1] de Bortoli D, Wrubleski E, Marczak RJ (2011) Hyperfit – Curve fitting software for incompressible hyper-elastic material models. Proceedings of COBEM 2011. 21st Brazilian Congress of Mechanical Engineering.

[2] Heung HL, et al. (2019) Robotic Glove with Soft-Elastic Composite Actuators for Assisting Activities of Daily Living. Soft Robot 6(2):289-304.

[3] Nikolov S, et. al (2016) Model-based design optimization of soft fiber-reinforced bending actuators. (Proc. IEEE Int. Conf. Manipulation, Manuf. Meas. Nanoscale (3M-NANO), Chongqing, China), pp 136-140.

[4] Connolly F, et al. (2017) Automatic design of fiber-reinforced soft actuators for trajectory matching. Proc Natl Acad Sci U S A 114(1):51–56.

[5] Wang Z, et al. (2017) Interaction Forces of Soft Fiber Reinforced Bending Actuators. IEEE/ASME Trans Mechatron. 22(2):717-727.

[6] Howell LL (2001) Compliant Mechanisms (John Wiley & Sons, Inc., New Jersey, U.S.A.)

[7] Lyle RC (1981) A performance test for assessment of upper limb function in physical rehabilitation treatment and research. Int J Rehabil Res 4(4):483–492.

[8] Lang CE, et al. (2006) Measurement of upper-extremity function early after stroke: properties of the action research arm test. Arch Phys Med Rehabil 87(12):1605–1610.

[9] Van der Lee JH, et al. (2002) Improving the action research arm test: a unidimensional hierarchical scale. Clin Rehabil 16(6):646–653.

[10] Ansari NN, et al. (2008) The interrater and intrarater reliability of the Modified Ashworth Scale in the assessment of muscle spasticity: limb and muscle group effect. NeuroRehabilitation 23(3):231-237

[11] Kamper DG, et al. (2006) Weakness is the primary contributor to finger impairment in chronic stroke. Arch Phys Med Rehabil 87(9):1262-1269.

[12] Haarman CJW, et al. (2018) Joint Stiffness Compensation for Application in the EXTEND Hand Orthosis (Proc IEEE RAS EMBS Int Conf Biomed Robot Biomechatron, Enschede, The Netherlands) pp 677-682

[13] Brokaw EB, et al. (2011) Hand Spring Operated Movement Enhancer (HandSOME): A portable, passive hand Exoskeleton for stroke rehabilitation. IEEE Trans Neural Syst Rehabil Eng 19(4):391-399

[14] Dionysian E, et al. (2005) Proximal interphalangeal joint stiffness: measurement and analysis. J Hand Surg Am 30(3):573–579
